# Study protocol of the German "Registry for the Detection of Late Sequelae after Radiotherapy in Childhood and Adolescence" (RiSK)

**DOI:** 10.1186/1748-717X-3-10

**Published:** 2008-04-21

**Authors:** Tobias Bolling, Andreas Schuck, Hildegard Pape, Christian Rube, Barbara Pollinger, Beate Timmermann, Rolf D Kortmann, Karin Dieckmann, Normann Willich

**Affiliations:** 1 Department of Radiotherapy, University Hospital of Münster, Germany; 2 Radiotherapy Facility, Memmingen, Germany; 3 Department of Radiotherapy, University Hospital of Düsseldorf, Germany; 4 Department of Radiotherapy, University Hospital of Homburg/Saar, Germany; 5 Department of Radiotherapy, University Hospital of Munich, Germany; 6 Department of Radiotherapy of the Paul-Scherrer-Institute, Villigen, Switzerland; 7 Department of Radiotherapy, University Hospital of Leipzig, Germany; 8 Department of Radiotherapy, University Hospital of Vienna, Austria

## Abstract

**Background:**

Late effects after radiotherapy in childhood and adolescence have mainly been characterized retrospectively with small patient numbers. However, these analyses are limited due to little information regarding organ dose levels in many cases. To overcome this limitation, the German Group of Paediatric Radiation Oncology (APRO) established the „Registry for the evaluation of late side effects after radiation in childhood and adolescence” (RiSK). The study protocol and the documentation forms are given in this publication.

**Methods/Design:**

Radiation parameters including detailed organ doses as well as toxicity evaluations are collected prospectively from centres all over Germany. Standardized documentation forms are used. These forms are given in an English and German version as additional files to this publication. Documentation is planned for all children who receive radiotherapy in one of the therapy trials of the "German Society of Paediatric Oncology and Haematology (GPOH)". The study started in a pilot phase in June 2001 in few centres. Since 2004 documentation has been performed all over Germany and is still on-going.

**Discussion:**

To our knowledge, "RiSK" is the only multi-centre study that evaluates radiation associated side effects prospectively with detailed information about organ dose levels. With ongoing recruitment and prolongation of follow-up powerful data will be obtained in a few years. A broad use and international cooperation are welcome.

## Background

Radiotherapy is of fundamental importance in paediatric oncology. Individual decisions for the use of ionizing radiation always have to consider the potential benefit and possible side effects. Whereas the benefit is no matter of major discussions, the risk for potential side effects like secondary malignancies and non-cancer health effects cannot be quantified based on sufficient data. Especially, there is a lack of information regarding dose-effect relationships of ionizing radiation. This is of special importance when ionizing radiation is used in childhood and adolescence due to potential higher vulnerability of growing tissue and longer expected life span [1-3]. For radiotherapy, late effects after treatment in childhood and adolescence have mainly been characterized retrospectively with small patient numbers [4-7]. Many of these analyses are limited due to little information about organ dose levels and older radiation techniques in some cases. Recently, the characterization of late effects after cancer therapy in childhood including chemotherapy, surgery and radiation has been of rising interest [8,9]. Several study groups in a variety of countries have developed strategies to characterize different aspects of late effects. In Germany, several study groups like the "Late Effects Surveillance System" (LESS) [10,11] or the working group "Quality of life" [12] have been established. The largest examination has been performed in the United States of America: The "Childhood Cancer Survivor Study" has been established to characterize retrospectively the health status of 5-year-survivors of childhood cancer. In these studies, more than 12,000 patients were evaluated by questionnaires regarding their health status [8]. For radiation exposure, this study cannot give very detailed information about organ-dose level associated late side effects due to quite insufficient data regarding radiation organ dose levels. So this approach turned out to be not sufficient in view of answering open questions regarding the risks of radiotherapy in childhood and adolescence.

## Methods/Design

To overcome the described lack of information and to receive detailed data regarding the risk of radiotherapy in childhood and adolescence, the German Group of Paediatric Radiation Oncology (APRO), a working group of the "German Society of Radiation Oncology" (DEGRO) and the "German Society of Paediatric Oncology and Haematology" (GPOH) established the „**R**eg**i**stry for the evaluation of late **s**ide effects after radiation in **c**hildhood and adolescence” (RiSK) [1-3]. The aim of this prospective multi-centre registry study is to evaluate radiation dose-effect relationships in organs and part of organs with special regard to late side effects. Documentation is planned for all children who receive radiotherapy in one of the therapy optimizing study trials of the GPOH. The documentation of radiotherapy is performed through local radiotherapists; the documentation forms are reviewed and collected in the study centre in Münster. In addition, the names of children who receive radiotherapy are collected from the therapy study centres of the GPOH study trials. The documentation forms of these patients are asked for in the departments of radiotherapy if no documentation has been performed. Parents' declarations of consent for data transfer have been integrated into most of the study protocols of the GPOH that are relevant in view of radiotherapy. Therefore additional declarations of consent are not necessary in Germany. The first toxicity evaluation, i.e. the documentation of acute toxicities, is scheduled at 6–8 weeks after the end of radiotherapy. Later on, toxicity evaluations have to be performed once a year. The evaluation of toxicities is performed either by the radiation oncologists or, due to problems in some departments, by the treating paediatric oncologists.

In a first step of RiSK, its structure was established and the documentation forms were developed. They include the evaluation of the treatment period, the fractionation schedule, the target volume(s) as well as the radiation techniques and doses including detailed information about organ dose levels. A study group (see Acknowledgements) of the APRO defined doses for each organ that should lead to documentation if these doses are reached. Beside the documentation of the maximum doses for some organs, the dose-volume histograms have to be characterized for the lungs, the heart, the liver and the kidneys. In case of the thyroid gland and the testes, dose measurements have to be performed. Surgery and chemotherapy are evaluated as well. Toxicity documentation is performed according to EORTC/RTOG criteria [13], adapted to paediatric values. The toxicity is characterized in 5 degrees (0 = no side effect, 4 = severe side effect) for each organ. There is no need for additional examinations in follow-up because the toxicity evaluations refer to the standard follow-up examinations. The study flow chart is given in Table 1. The documentation forms are available in the internet [14] and given as additional files to this publication in an English (see additional file 1) and a German (see additional file 2) version. The documentation for RiSK started in July 2001 with a pilot phase in few study centres. During this period, the documentation forms have been re-evaluated and adapted to the practical need. Since February 2004, the documentation has been performed all over Germany in a multi-centre way. The feasibility of "RiSK" has already been shown [1] and first results have recently been published [2,3].

**Table 1:** Study flow chart.

Timepoint	Documentation	Special needs
Treatment planning and radiotherapy	Basic data (see documentation form part 1)	
	Organ doses (see documentation form part 2)	1. Dose-volume-histogram for lungs, heard, liver and kidneys, if in radiation field. Therefore CT-based treatment planning is mandatory if irradiation is performed to the thorax or abdomen.
		2. Dosimetry of the thyroid gland if irradiation in head/neck area or mediastinum
		3. Dosimetry of the testes if irradiation is performed to the abdomen or to the upper part of the lower extremity
6–8 weeks after end of radiotherapy Follow-up examination	Documentation of maximal side effects that occured during or few weeks after radiotherapy according RTOG/EORTC criteria (see documentation form)	Only clinical examination
1 year after end of radiotherapy: follow-up examination	Evaluation of late sequelae according to RTOG/EORTC criteria (see documentation form)	Only clinical examination, blood values to be asked from paediatricians
2nd-10^th ^(if feasible) year after radiotherapy follow-up examination 1×/year	Evaluation of late sequelae according to RTOG/EORTC criteria (see documentation form)	Only clinical examination, blood values to be asked from paediatricians

The documentation forms have to be sent to the RiSK study trial center in Münster: RiSK-Study trial centre, Department of Radiotherapy, University Hospital of Münster, Albert-Schweitzer-Str. 33, D-48129 Münster, Germany, Phone: +49-251-83-47384, Fax: +49-251-83-47355, E-Mail: radtox@uni-muenster.de

## Discussion

Sufficient data regarding radiation associated late sequelae after radiotherapy in childhood and adolescence with special emphasis on organ dose-volume effects do not exist. Due to the lack of detailed organ dose evaluation in the pre-3-D-treatment planning era, retrospective analyses can only be performed with very uncertain estimations of the radiation doses at special organs. To our knowledge, "RiSK" is the only multi-centre study that evaluates radiation associated side effects prospectively with detailed information about organ dose levels. With ongoing recruitment and prolongation of follow-up powerful data will be obtained in a few years. The described study protocol and the documentation forms are open for the use by other study groups. To further increase the number of patients in order to obtain faster results, an international cooperation with other study groups evaluating radiation associated side effects after radiotherapy in childhood and adolescence is welcome.

## Abbreviations

APRO: Working Group Paediatric Oncology (*"Arbeitsgruppe Pädiatrische Radioonkologie"*); DEGRO: German Society of Radiation Oncology (*"Deutsche Gesellschaft für Radioonkologie"*); EORTC: European Organisation for Research and Treatment of Cancer; GPOH: Germany Society of Paediatric Oncology and Hematology (*"Gesellschaft für pädiatrische Onkologie und Hämatologie"*); RiSK: Registry for the evaluation of late sequelae after radiotherapy in childhood and adolescence (*"Register zur Erfassung von Spätfolgen nach Strahlentherapie im Kindes- und Jugendalter"*); RTOG: Radiation Therapy Oncology Group.

## Competing interests

The authors declare that they have no competing interests.

## Authors' contributions

TB is the study coordinator of RiSK, participated in the development of the study trial and drafted the manuscript. AS, HP, CR, BP, BT, RDK and KD participated in the design of the registry study and are involved in continuing optimization. NW is the study chairman of RiSK, developed the design of the registry study, is involved in continuing optimization and helped to draft the manuscript. All authors read and approved the final manuscript.

## Supplementary Material

Additional file 1 RiSK-documentation form English. This file is the English version of the original German RiSK documentation form. Click here for file

Additional file 2 RiSK-Dokumentationsbogen Deutsch. This file is the original German version of the RiSK documentation form. Click here for file
