# Supplementary material for: Study protocol of the German "Registry for the Detection of Late Sequelae after Radiotherapy in Childhood and Adolescence" (RiSK)
Source: Radiat Oncol. 2008 Apr 21;3:10. doi: 10.1186/1748-717X-3-10 (PMC2373793; doi:10.1186/1748-717X-3-10)
Supplement: Additional file 1 — RiSK-documentation form English. This file is the English version of the original German RiSK documentation form. [file 1748-717X-3-10-S1.pdf]

# RiSK

## RADIOTHERAPY – Documentation form

-

Primary therapy: ☐ Relapse therapy: ☐

Name, Surname: ..............................................................................................................

DOB:_____._____.__________ Sex: ☐ male ☐ female

Address:............................................................................................................................

Diagnosis: .........................................................................................................................

Therapy protocol: ..............................................................................................................

Department of Pediatric Oncology:....................................................................................
.................................................................................................................................
.................................................................................................................................
.................................................................................................................................

Department of Radiotherapy:.............................................................................................
.................................................................................................................................
.................................................................................................................................
.................................................................................................................................

Tumor location:..................................................................................................................

Target of Radiotherapy: .....................................................................................................
(For details see list on page 5)

Functional impairment within the treatment area before radiotherapy: ☐ no

☐ yes:.................................................................................................................................

...........................................................................................................................................

Functional impairment outside the treatment area before radiotherapy: ☐ no

☐ yes:.................................................................................................................................

...........................................................................................................................................

Height before start of radiotherapy (cm):_______ Height if seated before radiotherapy (cm) (in case of radiation of spinal axis):______

Weight before start of radiotherapy (kg):______________

Documentation forms should be sent back to the following address:
RiSK study trial center, Department of Radiotherapy, University Hospital of Münster, Albert-Schweitzer-Str.33, D-48129 Münster, Germany; Phone: +49-251-83-47384; Fax: +49-251-83-47355; Email: radtox@uni-muenster.de

**Radiation planning (in case of 2 localizations please use a second table)**

| | **Target volume I** | **Target volume II (e.g. shrinking field, boost)** | **Target volume III (e.g. shrinking field, boost)** |
|---|---|---|---|
| Irradiated organ area: | | | |
| | ☐single field<br>☐ opposing fields<br>☐> 2 fields | ☐single field<br>☐ opposing fields<br>☐> 2 fields | ☐single field<br>☐ opposing fields<br>☐> 2 fields |
| No. of treatment fields: | | | |
| Non-coplanar fields? | ☐ yes ☐ no | ☐ yes ☐ no | ☐ yes ☐ no |
| Coplanar fields? | ☐ yes ☐ no | ☐ yes ☐ no | ☐ yes ☐ no |
| Isodose plan? | ☐ yes ☐ no | ☐ yes ☐ no | ☐ yes ☐ no |
| CT/MRI – based? | ☐ yes ☐ no | ☐ yes ☐ no | ☐ yes ☐ no |
| 3- D planning? | ☐ yes ☐ no | ☐ yes ☐ no | ☐ yes ☐ no |
| Dose volume histogram? | ☐ yes ☐ no | ☐ yes ☐ no | ☐ yes ☐ no |
| | | | |
| Prescription according to ICRU 50 report? | ☐ yes ☐ no | ☐ yes ☐ no | ☐ yes ☐ no |
| Surrounding isodose: | % | % | % |
| Dosage on ½ diameter? | ☐ yes ☐ no | ☐ yes ☐ no | ☐ yes ☐ no |
| Dosage Depth: | cm | cm | cm |
| Other dosage system: | | | |
| Simulation? | ☐ yes ☐ no | ☐ yes ☐ no | ☐ yes ☐ no |
| Cobalt 60? | ☐ yes ☐ no | ☐ yes ☐ no | ☐ yes ☐ no |
| Linac- Photons? | ☐ yes ☐ no | ☐ yes ☐ no | ☐ yes ☐ no |
| Linac-Electrons:<br>Energy: | ☐ yes ☐ no<br>____ MeV | ☐ yes ☐ no<br>____ MeV | ☐ yes ☐ no<br>____ MeV |
| Neutrons? | ☐ yes ☐ no | ☐ yes ☐ no | ☐ yes ☐ no |
| Protons? | ☐ yes ☐ no | ☐ yes ☐ no | ☐ yes ☐ no |
| Mixed technique? | ☐ yes ☐ no | ☐ yes ☐ no | ☐ yes ☐ no |
| Bolus material? | ☐ yes ☐ no | ☐ yes ☐ no | ☐ yes ☐ no |
| Stereotact. irradiation? | ☐ yes ☐ no | ☐ yes ☐ no | ☐ yes ☐ no |

## Other irradiation techniques

☐ Brachytherapy:

☐endoluminal ☐ interstitial ☐ Flab-Technique ☐ Seed-Implantation

☐ Intraoperative Radiotherapy (IORT)

☐ Electrons ☐Brachytherapy (Flab)

☐ open radionuclides

☐ Positioning help (which one?) ☐ fixation mask ☐ Vacuum cushion ☐plaster cushion

☐ Sedation/short narcosis ☐ Stereotactic fixation ring

☐ other:..................……………………….......................

**Radiation planning (in case of 2 localizations please use a second table)**

| | **Target volume I** | **Target volume II** | **Target volume III** |
|---|---|---|---|
| Start of treatment: | | | |
| End of treatment: | | | |
| No. of treatment days: | | | |
| Treatment pauses: | ☐ yes , ☐☐ days<br>☐ no | ☐ yes , ☐☐ days<br>☐ no | ☐ yes , ☐☐ days<br>☐ no |
| Reason: | | | |
| Cumulative radiation dose in target volume (Gy): | | | |
| Single fraction dose (Gy): | | | |
| Fractionation schedule:: | x Gy /Week | x Gy /Week | x Gy /Week |

Notices:.........................................................................................................................

☐ Total body irradiation (TBI):

Date of treatment:...............................

| | | | | |
|---|---|---|---|---|
| Single dose (Gy) : | | | Lung dose: | |
| Fractionation schedule: | .......... x.......... Gy /Tag | | Liver dose: | |
| TBI total dose | | | Kidney dose: | |
| Dose / time: | | | | |

**<u>Has radiotherapy been performed earlier?</u>** ☐ no ☐yes

if yes, target volume:...................................... Dose:...................................Gy

Year of irradiation:..........................

Surgery in irradiated volume: ☐yes ☐ no ☐ unknown

performed before radiotherapy: ☐yes ☐ no

planned after radiotherapy: ☐yes ☐ no

Kind of Surgery:........................................................................................................

Chemotherapy before radiotherapy : ☐yes ☐ no ☐ unknown

Substances..................................................................................................................

.................................................................................................................................

Chemotherapy simultaneously to radiotherapy : ☐yes ☐ no ☐ unknown

Substances..................................................................................................................

.................................................................................................................................

Chemotherapy after radiotherapy : ☐yes ☐ no ☐ unknown

Substances..................................................................................................................

.................................................................................................................................

☐ Therapy associated acute toxicity EORTC / RTOG Grade 3 or 4 during radiotherapy:

a)................................................................................................................................................

b )...............................................................................................................................................

c )................................................................................................................................................

.......................... ...................................... .......................................................

Date Name of Physician Signature / stamp

# List of treatment volumes

| | | | | |
|---|---|---|---|---|
| 1 | Skin | | | **Abdomen** |
| 2 | Total body | | 42 | abdomen |
| 3 | Half body | | 43 | liver |
| | **CNS** | | 44 | liver hilus |
| 81 | craniospinal axis | | 45 | splen, splen hilus |
| 4 | whole brain | | 46 | stomach |
| 5 | cerebrum | | 47 | paraaortal lymph nodes |
| 6 | cerebellum | | 48 | pankreas |
| 7 | brainstem | | 49 | kidney |
| 8 | hypophysis | | 50 | adrenal gland |
| 9 | pinealis | | 51 | iliacal lymph nodes |
| 10 | suprasellar region | | 52 | inguinal lymph nodes |
| 11 | bones of skull | | 53 | small pelvis |
| 12 | ethmoidal sinus | | 54 | pelvic wall |
| 13 | orbita | | 55 | rectum |
| 14 | retrobulbic region | | 56 | pre-sacrum region |
| | **Head/Neck** | | 57 | anal |
| 15 | cheek | | 58 | bladder |
| 16 | Waldeyer’s ring in the throat | | 59 | prostate |
| 17 | tonsilla | | 60 | testicles |
| 18 | thyreoid gland | | 61 | uterus |
| 19 | epipharyngeal region | | 62 | parametrium |
| 20 | mesopharyngeal region | | 63 | ovary |
| 21 | hypophayngeal regon | | 64 | vagina |
| 22 | maxilla | | 65 | vulva |
| 23 | mandibula | | | **Spinal cord + spine** |
| 24 | sinus maxillaris | | 66 | myelon |
| 25 | tongue | | 67 | cervical spine |
| 26 | mouth soil | | 68 | thoracic spine |
| 27 | larynx | | 69 | lumbal spine |
| 28 | submental lymph nodes | | 70 | sacrum |
| 29 | cervical lymph nodes | | 71 | paravertebral region |
| 30 | supra-/infraclavicular LN | | | **Extremities** |
| | **Thorax** | | 72 | shoulder / Neck |
| 31 | mediastinum | | 73 | upper arm |
| 32 | hilomediastinal | | 74 | forearm |
| 33 | hilus | | 75 | hand |
| 34 | esophagus | | 76 | hip |
| 35 | heart | | 77 | upper leg |
| 36 | whole lung | | 78 | lower leg |
| 37 | central lung | | 79 | foot |
| 38 | peripher lung | | | |
| 39 | chest wall | | 80 | other: |
| 40 | mamma | | | |
| 41 | axilla | | | |

# RiSK

## RADIOTHERAPY – DOCUMENTATION FORM

## - Organ dose levels -

**Guidelines for documentation**

- **On the following pages only radiation doses of organs / areas in the radiation field should be given. Radiation dose exposures outside the radiation fields should be given only for ovaries / testicles and the thyroid gland.**

- **For thyroid gland and testicles dose measurements (e.g. by thermoluminescence dosimetry) are planned.** If possible, several measurements points should be spread over the organ. In case of one single measurement point this point should be placed at a representative point in the middle of the organ.

- **For lung, heard, liver and kidneys the organ dose levels have to be taken from dose-volume-histograms of the treatment plan.**

- **D-max: maximal punctual dose in the organ**
  **D-min: minimal punctual dose in the organ**

- **I. Part: Documentation of doses at bones, soft tissues and joints**
  **II. Part: Documentation of organs, listed from cranial to caudal.**

# *I. Part*

## Bone marrow

| **Please mark the field contour** |
|---|

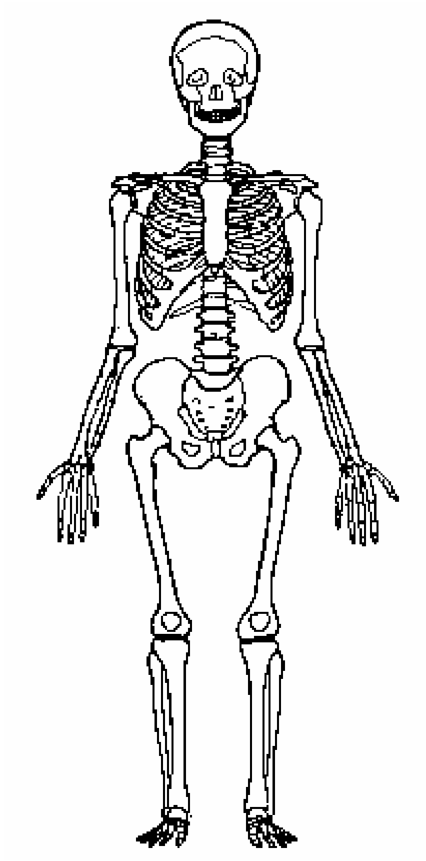

(Please mark the field contour)

## Spine and spinal cord

| **Dose documentation and toxicity follow-up for vertebrae and spinal cord for doses > 10 Gy** |
|---|

**Irradiated vertebrae**: from...............................to................................

D-max in irradiated vertebrae: ...............Gy D-min in irradiated vertebrae:............Gy

**Dose gradient below 20Gy in the vertebrae?** ☐ yes ☐ no

**Irradiated segments of spinal cord**: from..............................to................................

D-max in irradiated segments: ...............Gy D-min in irradiated segments:............Gy

## BONES AND SOFT TISSUE

**Dose documentation and toxicity follow up if irradiated with ≥ 10 Gy**

| | D-max (Gy) | Whole bone in target volume? |
|---|---|---|
| Bones of skull | | ——— |
| Ribs right side | | ——— |
| Ribs left side | | ——— |
| Pelvis right | | |
| Pelvis left | | |
| Sacrum | | |

| | D-max (Gy) | Whole bone in PTV? | | Proximal epiphysis interstice in field? | | | Distal epiphysis interstice in field? | | |
|---|---|---|---|---|---|---|---|---|---|
| | | yes | no | yes | partial | no | yes | partial | no |
| Humerus right | | | | | | | | | |
| Radius right | | | | | | | | | |
| Ulna right | | | | | | | | | |
| Hand right | | | | —— | —— | —— | —— | —— | —— |
| Femur right | | | | | | | | | |
| Tibia right | | | | | | | | | |
| Fibula right | | | | | | | | | |
| Foot right | | | | —— | —— | —— | —— | —— | —— |
| | | | | | | | | | |
| Humerus left | | | | | | | | | |
| Radius left | | | | | | | | | |
| Ulna left | | | | | | | | | |
| Hand left | | | | —— | —— | —— | —— | —— | —— |
| Femur left | | | | | | | | | |
| Tibia left | | | | | | | | | |
| Fibula left | | | | | | | | | |
| Foot left | | | | —— | —— | —— | —— | —— | —— |

**Complete circumference of the extremity in field:** ☐ yes (without lymphatic space) ☐ no

## JOINTS

**Dose documentation and toxicity follow-up if irradiated with ≥ 20 Gy**

**Joints in radiation field:** ☐ no ☐ yes

| | Right (Gy) | Left (Gy) |
|---|---|---|
| Shoulder | | |
| Elbow | | |
| Hand (radio-carpal) | | |
| Hip | | |
| Knee | | |
| Ankle joint | | |

## *II. Part (Organ dose levels)*

**CNS**

**Dose documentation and toxicity follow up if in target volume**

☐ **Whole brain irradiation (CNS prophylaxis / CNS- manifestation in case of ALL / AML)**

Planned dose:..............Gy

Dosimetry thyroid gland: measured total dose:......../……../……../……../……..Gy
(or dose evaluation from treatment plan), alternative: distance between field border and thyroid gland: ………..cm
(**no further documentation necessary!!**)

☐ **Whole brain irradiation** planned dose:............ Gy

☐ **cranio-spinal axis radiation** planned dose:............ Gy

Boost spinal: ☐ no ☐ yes: cumulative dose:..........Gy
Myelon segment:.…..................

Boost cerebral: ☐ no ☐ yes: cumulative dose…........Gy
region:............................

☐ **Radiation of parts of the brain:**

☐ < 20 Gy → no documentation necessary

☐ > 20 Gy

| | D-max (Gy) | D-min (Gy) |
|---|---|---|
| Hypophysis | | |
| Optic chiasm | | |
| Brainstem | | |

**Spinal cord: see Part I**

**ORBITA**

**Dose documentation and toxicity follow-up for any dose given**

| | D-max (Gy) | Whole organ in PTV? | |
|---|---|---|---|
| | | yes | no |
| Eye right | | | |
| Lens right | | ———— | |
| Tear gland right | | | |
| Optic nerve right | | | |

| | D-max (Gy) | Whole organ in PTV? | |
|---|---|---|---|
| | | yes | no |
| Eye left | | | |
| Lens left | | ———— | |
| Tear gland left | | | |
| Optic nerve left | | | |

**EYE**

**Dose documentation and toxicity follow-up for any given dose**

| | D-max (Gy) |
|---|---|
| Middle ear ri. | |
| Inner ear ri. | |

| | D-max (Gy) |
|---|---|
| Middle ear l. | |
| Inner ear left | |

**Parallel chemotherapy with Cisplatin:**
☐ yes ☐ no

## SALIVARY GLANDS

**Dose documentation and toxicity follow-up if irradiated with ≥ 20 Gy**

| | D-max (Gy) | Complete in field? | |
|---|---|---|---|
| Gl. parotis ri. | | ☐yes | ☐no |
| Gl. submandibularis ri. | | ☐yes | ☐no |
| | | | |
| Gl. parotis le. | | ☐yes | ☐no |
| Gl. submandibularis le. | | ☐yes | ☐no |

## TEETH / JAW

**Dose documentation and toxicity follow-up for any given dose.**

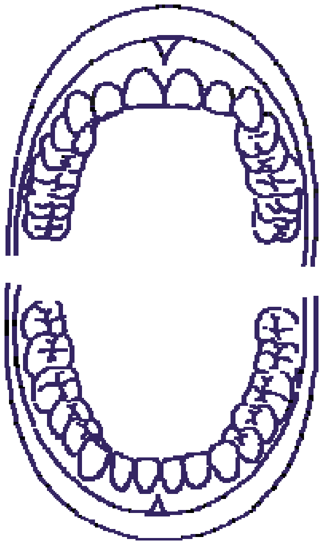

Please mark the irradiated region!

Jaw joint left in treatment field? ☐ no ☐ yes, dose:..................Gy

Jaw joint right in treatment field? ☐ no ☐ yes, dose:..................Gy

## THYROID GLAND

**Dose documentation and toxicity follow-up for any given dose.**
**<u>Dosimetric evaluation</u> of scattered dose, e.g. in case of brain or mediastinal irradiation; alternative: distance of organ from field border.**

☐ **Thyroid gland in target volume** D-max:...................Gy

Whole organ in target volume: ☐ No ☐ Yes

☐ **Thyroid gland not in target volume**
(in case of head/neck or mediastinal irradiation / cranio-spinal axis irradiation)
(several measurement points)

Dosimetry: ☐ yes → measured total dose:……../……../……../……../……..Gy
(or dose evaluation from treatment plan)
☐ no→ Distance to field border:...................cm

## Larynx

**Dose documentation and toxicity follow-up in case of irradiation ≥ 20 Gy.**

D-max: ............Gy

Part of irradiated organe volume ≥ 20 Gy: ☐ < 1/3 ☐ 1/3 – 2/3 ☐ > 2/3 ☐ complete

## BREAST

**Dose documentation and toxicity follow-up for any given dose.**

| | D-max (Gy) | Whole breast in PTV | |
|---|---|---|---|
| | | Yes | No |
| Breast right | | | |
| | | | |
| Breast left | | | |

Symmetric Irradiation of both breast: ☐ yes ☐ no

## ESOPHAGUS

**Dose documentation and toxicity follow-up in case of partial irradiation ≥ 40 Gy.**

Part of esophagus with dose ≥ 40 Gy
from.......................... to ............................ (Documentation of localization according to vertebrae)
D-max in irradiated segment: .................... Gy

## PANCREAS

**Dose documentation and toxicity follow-up in case of partial irradiation ≥ 20 Gy**

D-max:...................Gy

Part of irradiated organ volume ≥ 20 Gy:
☐ < 1/3
☐ 1/3 – 2/3
☐ > 2/3
☐ complete

## URINARY BLADDER

**Dose documentation and toxicity follow-up if in target volume.**

D-max:...................Gy; Urinary bladder complete in PTV: ☐ no ☐ yes

# DOSE-VOLUME-HISTOGRAM (DVH)

## HEART

**Dose documentation and toxicity follow-up if irradiated with ≥ 15 Gy. Evaluation also in case of infra-diaphragmal irradiation.**

## LUNG

**Dose documentation and toxicity follow-up in case of whole lung irradiation ≥ 10 Gy or partial lung irradiation ≥ 18 Gy. Dose volume histogram!**

## LIVER

**Dose documentation and toxicity follow-up in case of irradiation ≥ 1/3 of the liver and/or partial liver irradiation ≥ 20 Gy. Dose volume histogram!**

## Kidneys

**Dose documentation and toxicity follow-up if ≥ 1/2 of one kidney is irradiated and/or a partial kidney dose ≥ 12 Gy has been reached. Dose volume histogram!**

Alternative: DVH as a copy or fill out the table.

| Dose | Heart | Lung left | Lung right | Whole lung | Liver | Kidney left | Kidney right |
|---|---|---|---|---|---|---|---|
| > 5 Gy | % | % | % | % | % | % | % |
| >10 Gy | % | % | % | % | % | % | % |
| >15 Gy | % | % | % | % | % | % | % |
| >20 Gy | % | % | % | % | % | % | % |
| >30 Gy | % | % | % | % | % | % | % |
| >40 Gy | % | % | % | % | % | % | % |
| >50 Gy | % | % | % | % | % | % | % |
| >60 Gy | % | % | % | % | % | % | % |

**Pre-existing functional impairment of heart, lungs, liver or kidneys:**

☐ no ☐ yes:

…......................................................................................................................................

………………..........................................................................................................................

## BOWEL

**Dose documentation and toxicity follow-up in case of partial abdomen irradiation ≥ 10 Gy.**

**Small intestine:** D-max:..................Gy

Part of irradiated volume ≥ 20 Gy:

☐ < 1/3

☐ 1/3 – 2/3

☐ > 2/3

☐ complete

**Colon:** D-max:...............Gy

Part of irradiated volume ≥ 20 Gy:

☐ < 1/3

☐ 1/3 – 2/3

☐ > 2/3

☐ complete

..

## VAGINA / UTERUS

**Dose documentation and toxicity follow-up if in PTV.**

| | Dose in PTV |
|---|---|
| Vagina | |
| Cervix | |
| Uterus | |

Part of Vagina in treated volume:

☐ Partial irradiation ☐ complete

## OVARIES

Dose documentation and toxicity follow-up for any given dose.

**Ovary right:** ☐ in radiation field, dose: → ................Gy
☐ scattered dose: → ................Gy (Dose evaluation from treatment plan)

**Ovary left:** ☐ in radiation field, dose: → ................Gy
d☐ scattered dose: → ................Gy (Dose evaluation from treatment plan)

**Ovary transposition:** ☐ no ☐ yes →**moved to:** .........................
..................................................................

## TESTICLES

**Dose documentation and toxicity follow-up for any dose given to the abdomen. <u>Dosimetric evaluation of scattered dose in case of irradiation of pelvis or upper leg.</u>**

**Irradiation with testicle capsule?** ☐ yes ☐ no

**Testicle right:**
☐ in radiation field→ .........Gy
☐ Scattered dose: Dosimetry
☐ yes→total dose ........../........../........../........../...........Gy
☐ no→distance from field border (cm):.........

**Testicle left:**
☐ in radiation field → .........Gy
☐ Scattered dose: Dosimetry
☐ yes→ total dose: ........../........../........../...........Gy
☐ no→distance from field border: (cm):……………..

| ................. | .................................................... | .................................................................. |
|---|---|---|
| **Date:** | **Name of Radiotherapist** (print letters) | **Signature / Stamp of institution** |

| No. | Date . . | **Max. acute side effects after radiotherapy in childhood (adapted from RTOG/EORTC)** | Pat.name, DOB: |
|---|---|---|---|

| | | | | | |
|---|---|---|---|---|---|
| **General status, Karnofsky-Index** | **0** normal<br>KI: 100% | **1** Mild reduced activity,<br>KI: 70–80% | **2** Reduced activity and power, KI: 50–60% | **3** Severe reduced activity and power, KI: 30–40% | **4** Vital threatened,<br>KI: < 30% |
| **Bone marrow** | **0** | **1** | **2** | **3** | **4** |
| Leucocytes (N/µl) | ≥ 4.000 | 3.000 - < 4.000 | 2.000 - < 3.000 | 1.0 - < 2.000 | < 1.000 |
| Neutrophils (N/µl) | ≥ 2.000 | < 2.000 - 1.500 | < 1.500 - 1.000 | < 1.000 - 500 | <500 / Sepsis |
| Thrombocytes (*$10^3$/µl) | ≥ 100 | 75 - < 100 | 50 - < 75 | 10,0 - < 50 | < 10,0 |
| Hemoglobin (g/dl) | normal | 10,0 - < N | 8,0 - < 10,0 | 6,5 - < 8,0 | < 6,5 |
| **Skin / Subcutis** | **0** normal | **1** Follicular, faint or dull erythema/epilation/dry desquamation/decreased sweating | **2** Tender or bright erythema, patchy moist desquamation/moderate edema; (local therapy necessary) | **3** Confluent, moist desquamation other than skin folds, pitting edema (intensive therapy necessary) | **4** Ulceration, hemorrhage or necrosis, (operative therapy necessary) |
| **Mucous membrane** | **0** normal | **1** Injection/may experience mild pain not requiring analgesic. | **2** Patchy mucositis that may produce an inflammatory sero-sanguinous discharge/may experience moderate pain requiring analgesia | **3** Confluent fibrinous mucositis/may include severe pain requiring narcotic | **4** Ulceration, hemorrhage or necrosis |
| **Salivary glands** | **0** normal | **1** Mild mouth dryness/ slightly thickened saliva/may have slightly altered taste | **2** Moderate to complete dryness/thick, sticky saliva / markedly altered taste. | **3** Complete dryness | **4** Acute salivary gland necrosis |
| **Pharynx/ Esophagus** | **0** normal | **1** Mild dysphagia or odynophagia/may require topical anesthetic or non-narcotic analgesics/may require soft diet | **2** Moderate dysphagia or odynophagia/may require narcotic analgesics/may require puree or liquid diet | **3** Severe dysphagia or odynophagia with dehydration or weight loss >15% from pre-treatment baseline, requiring N-G feeding tube, i.v. fluids or hyperalimentation | **4** Complete obstruction, ulceration, perforation, fistula |
| **Larynx** | **0** normal | **1** Mild or intermittent hoarseness/cough not requiring antitussive / erythema of mucosa | **2** Persistent hoarseness but able to vocalize, sore throat, patchy fibrinous exudates or mild arytenoids edema not requiring narcotic/cough requiring antitussive | **3** Whispered speech, throat pain requiring narcotic / confluent fibrinous exudates, marked arytenoids edema | **4** Marked dyspnea, stridor or hemoptysis with tracheostomy or intubation necessary |
| **Lung** | **0** no change | **1** Mild symptoms of dry cough or dyspnea on exertion | **2** Persistent cough requiring narcotic, antitussive agents / dyspnea with minimal effort but not at rest | **3** Severe cough unresponsive to narcotic antitussive agent or dyspnea at rest / clinical or radiological evidence of acute pneumonitis/intermittent oxygen or steroids may be required | **4** Severe respiratory insufficiency / continuous oxygen or assisted ventilation |
| **Heart** | **0** Normal | **1** Asymptomatic but objective evidence of ECG changes or pericardial abnormalities without evidence of other heart disease | **2** Symptomatic with ECG changes and radiological findings of congestive heart failure or pericardial disease/no specific treatment required | **3** Congestive heart failure, angina pectoris, pericardial disease responding to therapy | **4** Congestive heart failure, angina pectoris, pericardial disease, arrhythmia not responsive to non-surgical measures |
| **Arrhythmia** | **0** none | **1** Asymptomatic, no therapy | **2** recurrent / persistent, no therapy | **3** Therapy required | **4** Hypotension, ventricular Arrhythmia, defibrillation |
| **Cardiac function** | **0** normal | **1**Asymptomat. EF ↓ (at rest) > = 10 % but < 20 % of baseline value | **2** Asymptomat., but EF ↓ (at rest) under upper limit for EF (work) or EF ↓ > = 20 % of baseline value | **3** Mild CHF, compensated by therapy | **4** severe / refractive CHF or need for intubation |
| **Echocardio: LV-SF %** | **0** > = 30 | **1** > = 24 - < 30 | **2** > = 20 - < 24 | **3** > 15 - < 20 | **4** < = 15 |
| **Upper G.I.** | **0** normal | **1** Anorexia with <=5% weight loss from pre-treatment baseline / nausea not requiring antiemetics / abdominal discomfort not requiring parasympatholytic drugs or analgesics | **2** Anorexia with <=15% weight loss from pre-treatment baseline / nausea &/or vomiting requiring antiemetics/ abdominal pain requiring analgesics | **3** Anorexia with >15% wt loss from pre-treatment baseline or requiring N-G tube or parenteral support. Nausea or vomiting requiring tube or parenteral support / abdominal pain, severe despite medication / hematemesis or melena / abdominal distention (flat plate radiograph demonstrates distended bowel loops) | **4** Ileus, subacute or acute obstruction, perforation, GI bleeding requiring transfusion, abdominal pain requiring tube decompression or bowl diversion |

| | | | | | |
|---|---|---|---|---|---|
| **Lower G.I. including pelvis** | **0** normal | **1** Increased frequency or change in quality of bowel habits not requiring medication/rectal discomfort not requiring analgesics. | **2** Diarrhea requiring parasympatholytic drugs /mucous discharge not necessitating sanitary pads/rectal or abdominal pain requiring analgesics. | **3** Diarrhea requiring parenteral support/severe mucous or blood discharge necessitating sanitary pads/abdominal distention (flat plate radiograph demonstrates distended bowel loops) | **4** Acute or subacute obstruction, fistula or perforation; GI bleeding requiring transfusion; abdominal pain or tenesmus requiring tube decompression or bowel diversion |
| **Liver** | **0** normal | **1** Mild lassitude; nausea, dyspepsia, slightly abnormal liver function, | **2** Moderate symptoms; some abnormal liver function tests; serum albumin normal | **3** Disabling hepatic insufficiency; liver function tests grossly abnormal; low albumin; edema or ascites | **4** Necrosis / Hepatic coma or encephalopathy |
| **Bilirubin** | **0** age norm (N) | **1** > N – 1,5 x N | **2** > 1,5 – 3,0 x N | **3** > 3,0 – 10,0 x N | **4** > 10,0 x N |
| **S-GOT / S-GPT** | **0** age norm (N) | **1**> N – 2,5 x N | **2** > 2,5 – 5,0 x N | **3** > 5,0 – 20,0 x N | **4** > 20,0 x N |
| **Genitourinary (and kidneys)** | **0** normal | **1** Frequency of urination or nocturia twice pre-treatment habit / dysuria, urgency not requiring medication | **2** Frequency of urination or nocturia that is less frequent than every hour. Dysuria, urgency, bladder spasm requiring local anesthetic | **3** Frequency with urgency and nocturia hourly or more frequently/dysuria, pelvic pain or bladder spasm requiring regular, frequent narcotic/gross hematuria with/without clot passage | **4** Hematuria requiring transfusion/acute bladder obstruction not secondary to clot passage, ulceration, or necrosis. |
| **Creatinin (mg%)** | **0** age norm | **1** > N – 1,5 x N | **2** > 1,5 – 3,0 x N | **3** > 3,0 – 6,0 x N | **4** > 6,0 x N |
| **Creatinin-Clearence** | > 90 | 60 – 89 | 40 –59 | 20 – 39 | < 19 |
| **Proteinuria (g/i)** | none | < 3 | 3 – 10,0 | > 10,0 | Nephrotic Syndrome |
| **Hematuria** | none | microscopic | macroscopic without clot passage | macroscopic with clot passage | Transfusion necessary |
| **Urethra and bladder** | **0** normal | **1** mild mucosa atrophy, small area of teleangi-ectasiae, microscopic hematuria, mild strengthened urinary urge | **2** moderate altered urinary urge (>6 times / day), generalized teleangiectasiae, sometimes macrohematuria, | **3** severe altered urinary urge, (>1/hour), dysuria, often hematuria | **4** necrosis, perforation, fistula, severe bladder contracture |
| **Bones** | **0** normal | **1** no symptoms, no growth retardation, mild reduced bone density | **2** Moderate pain or tension; moderate growth impairment, irregular bone sclerosis | **3** severe pain or tension, complete growth inhibition, thick bone sclerosis | **4** necrosis, osteoradionecrosis, spontaneous fracture |
| **Joints** | **0** normal | **1** mild joint stiffness, no symptoms or mild function impairment | **2** Moderate joint stiffness, intermediate joint pain, moderate function impairment | **3** severe joint stiffness, severe joint pain, severe function impairment | **4** necrosis, complete ancylosis, complete function impairment |
| **CNS (brain)** | **0** normal | **1** complete functional status with mild neurological impairment; no specific therapy necessary | **2** moderate neurological impairment; supportive care and medication (incl. steroids or anticonvulsives) necessary | **3** severe neurological impairment, high-dose steroids necessary, need for hospitalization | **4** life-threatening neurological impairment, >3 seizures per week despite of medication |
| **Peripheral nerves incl. spinal cord** | **0** normal | **1** mild L'Hermitte's syndrome, paraesthesiae | **2** marked L'Hermitte's syndrome with paraesthesiae and muscle weakness | **3** objective segmental neurological function loss, with paresis or paraesthesiae | **4** mono-, para- or quadraplegia |
| **Eyes** | **0** no change | **1** Mild conjunctivitis with or without scleral injection/increased tearing | **2** Moderate conjunctivitis with or without keratitis requiring steroids &/or antibiotics/dry eye requiring artificial tears/iritis with photophobia | **3** Severe keratitis with corneal ulceration/objective decrease in visual acuity or in visual fields/ acute glaucoma/ panophthalmitis | **4** Loss of vision (unilateral or bilateral) |
| **Ears** | **0** no change or baseline | **1** Mild external otitis with erythema, pruritis, secondary to dry desquamation not requiring medication. Audiogram unchanged from baseline. | **2** Moderate external otitis requiring topical medication / serous otitis medius / hypoacusis on testing only | **3** Severe external otitis with discharge or moist desquamation / symptomatic hypoacusis/tinnitus, not drug related | **4** Deafness |

**Adapted from:** Cox JD, Stetz J, Pajak TF. Toxicity criteria of the Radiation Therapy Oncology Group (RTOG) and the European Organization for Research and Treatment of Cancer (EORTC). Int J Radiat Oncol Biol Phys 1995;31:1341-1346.

………………………………………………

Name of Physician Signature / Stamp of Department

| No. | Date . . | Late side effects after radiotherapy in childhood (RTOG/EORTC) | Pat.name, DOB |
|---|---|---|---|

| Status | Complete remission | Progression | Secondary malignancy | Death | Date of death: |
|---|---|---|---|---|---|
| **General status, Karnofsky-Index** | **0** normal<br>KI: 100% | **1** Mild reduced activity,<br>KI: 70–80% | **2** Reduced activity and power,<br>KI: 50–60% | **3** Severe reduced activity and power,<br>KI: 30–40% | **4** Life threatened,<br>KI: < 30% |
| **Hb** *(g/dl)* | **0** age norm | **1** 10,0 - < N | **2** 8 – < 10,0 | **3** 6,5 – < 8,0 | **4** < 6,5 |
| **Leukocytes** *(N/µl)* | **0** ≥ 4.000 | **1** 3.000 – < 4.000 | **2** 2.000 – < 3.000 | **3** 1.000 – < 2.000 | **4** < 1.000 |
| **Thrombocytes** *(1000/µl)* | **0** ≥ 100 | **1** 75 – < 100 | **2** 50 – < 75 | **3** 10 - < 50 | **4** < 10 |
| **Skin** | **0** normal | **1** Slight atrophy, pigmentation change, some hair loss | **2** Patch atrophy, moderate teleangiectasia (≤ 50%), total hair loss | **3** Marked atrophy, gross teleangiektasia (> 50%) | **4** ulceration, necrosis, (surgical intervention necessary) |
| **Subcutaneous tissue** | **0** normal | **1** mild asymptomatic fibrosis, without contractures, slight reduced subcutaneous fat | **2** moderate asymptomatic fibrosis with ≤ 10% linear contracture, moderate reduced subcutaneous fat | **3** severe (symptomatic) fibrosis with ≥ 10% linear contractures, severe reduced subcutaneous fat | **4** ulceration, necrosis, (surgical intervention necessary) |
| **Mucous membrane** | **0** normal | **1** Slight atrophy or dryness | **2** moderate atrophy and teleangiectasia, reduced production of mucous | **3** Marked atrophy and teleangiectasia, loss of mucous production | **4** ulceration, necrosis, (surgical intervention necessary) |
| **Salivary glands** | **0** normal | **1** Slight dryness, good response on stimulation (normal food possible) | **2** moderate dryness of mouth, poor response on stimulation (much fluid, pulpy food) | **3** complete dryness of mouth, no response on stimulation (no solid food, fluid food) | **4** Fibrosis (complete atrophy) (parenteral nutrition, PEG) |
| **Pharynx/ Esophagus** | **0** normal | **1** mild fibrosis, slight dysphagia regarding solid food, no pain at swallow (normal nutrition) | **2** moderate fibrosis, no normal nutrition, pulpy food, perhaps dilatation necessary | **3** severe fibrosis (or dysphagia), only fluids possible, dilatation necessary, pain at swallow | **4** necrosis, perforation, fistula (surgical intervention necessary or PEG/ parenteral nutrition) |
| **Larynx** | **0** normal | **1** (mild) hoarseness (or cough), mild laryngeal edema | **2** moderate hoarseness or cough, moderate laryngeal edema, chondritis (symptomatic therapy) | **3** severe hoarseness, severe laryngeal edema, massive chondritis, intensive local therapy, analgesics | **4** necrosis, (massive dyspnoea and stridor, ulceration, intubation or tracheotomy |
| **Lung** | **0** normal<br>pO2: > 85;<br>pCO2 < 40 | **1** no or mild symptoms (dry cough); few radiological signs<br>(mild exercise-induced shortness of breath)<br>pO2:71-85; pCO2: 41-50 | **2** moderate symptomatic lung fibrosis or pneumonitis (massive cough); ,mild fever; radiological signs; (moderate exercise-induced shortness of breath)<br>pO2:61-70; pCO2: 51-60 | **3** Severe symptomatic lung fibrosis or pneumonitis, massive radiological signs; (severe shortness of breath)<br>pO2:51-60; pCO2: 61-70<br>(intensive medication) | **4** Massive respiratory insufficiency; permanent O2-application and controlled ventilation<br>pO2 < 50; pCO2 > 70<br>(intensive care necessary) |
| **Heart** | | | | | |
| **Arrhythmia** | **0** none | **1** Asymptomatic, no therapy | **2** recurrent / persistent, no therapy | **3** Therapy required | **4** Hypotension, ventricular Arrhythmia, defibrillation |
| **Cardiac function** | **0** normal | **1**Asymptomat. EF ↓ (at rest) > = 10 % but < 20 % of baseline value | **2** Asymptomat., but EF ↓ (at rest) under upper limit for EF (work) or EF ↓ > = 20 % of baseline value | **3** Mild CHF, compensated by therapy | **4** severe / refractive CHF or need for intubation |
| **Echocardio: LV-SF %** | **0** > = 30 | **1** > = 24 - < 30 | **2** > = 20 - < 24 | **3** > 15 - < 20 | **4** < = 15 |
| **Upper G.I.** | **0** normal | **1** Mild anorexia (≤ 5% weight loss); nausea; (once vomiting); mild abdominal pain;: no specific therapy | **2** moderate anorexia (≤ 15% weight loss); nausea or vomiting (2-5x), antiemetics necessary; moderate abdominal pain: mild parasympatholytics or analgesics necessary | **3** Massive anorexia (> 15% weight loss); nausea and vomiting (6-10x); NG-/PEG-probe or parenteral nutrition necessary; severe abdominal pain despite medication; hematemesis, melena; massive meteorism (X-Ray: extended bowel loops) | **4** necrosis; perforation, fistula; complete obstruction (ileus); GI-bleeding; PEG or parenteral nutrition, transfusions, surgery necessary |
| **Lower G.I: Bowel and colon** | **0** normal | **1** Mildly reduced stool consistence, mild spasms; stool ≤ 5x/day; mild slime or blood loss | **2** Markedly reduced stool consistence, spasms; stool > 5x/day; moderate slime and blood loss | **3** Massive heightened stool frequency, bowel obstructions needing surgical intervention, ileus or bleeding | **4** necrosis; perforation, fistula; (other life-threatening bowel complications) |

| | | | | | |
|---|---|---|---|---|---|
| **Liver** | **0** normal | **1** mild lassitude, nausea, dyspepsia | **2** moderate symptoms (tiredness, nausea, dyspepsia) | **3** severe liver insufficiency | **4** liver necrosis, hepatic coma or encephalopathy; (parenteral nutrition, portocaval shunt necessary) |
| **Bilirubin** | **0** age norm (N) | **1** > N – 1,5 x N | **2** > 1,5 – 3,0 x N | **3** > 3,0 – 10,0 x N | **4** > 10,0 x N |
| **S-GOT / S-GPT** | **0** age norm (N) | **1**> N – 2,5 x N | **2** > 2,5 – 5,0 x N | **3** > 5,0 – 20,0 x N | **4** > 20,0 x N |
| **Creatinin (mg%)** | **0** age norm | **1** > N – 1,5 x N | **2** > 1,5 – 3,0 x N | **3** > 3,0 – 6,0 x N | **4** > 6,0 x N |
| **Creatinin-Clearence** | > 90 | 60 – 89 | 40 –59 | 20 – 39 | < 19 |
| **Proteinuria (g/i)** | none | < 3 | 3 – 10,0 | > 10,0 | Nephrotic Syndrome |
| **Hematuria** | none | microscopic | macroscopic without clot passage | macroscopic with clot passage | Transfusion necessary |
| **Urethra and bladder** | **0** normal | **1** mild mucosa atrophy, small area of teleangi-ectasia, microscopic hematuria, mild strengthened urinary urge | **2** moderate altered urinary urge (>6 times / day), generalized teleangiectasia, sometimes macrohematuria, | **3** severe altered urinary urge, (>1/hour), dysuria, often hematuria | **4** necrosis, perforation, fistula, severe bladder contracture |
| **Bones** | **0** normal | **1** no symptoms, no growth retardation, mild reduced bone density | **2** Moderate pain or tension; moderate growth impairment, irregular bone sclerosis | **3** severe pain or tension, complete growth inhibition, thick bone sclerosis | **4** necrosis, osteoradionecrosis, spontaneous fracture |
| **Joints** | **0** normal | **1** mild joint stiffness, no symptoms or mild function impairment | **2** Moderate joint stiffness, intermediate joint pain, moderate function impairment | **3** severe joint stiffness, severe joint pain, severe function impairment | **4** necrosis, complete ancylosis, complete function impairment |
| **Function of irradiated extremity** | **0** normal | **1** mild function impairment, normal daily activities possible. | **2** moderate functional impairment at daily activities. | **3** Severe functional impairment. | **4** Loss of function |
| **Brain, Central nervous system** | **0** normal | **1** Mild headache, mild lethargia (mild neurological impairment) | **2** Moderate headache, obvious lethargia (somnolence < 50% / day); moderate neurological impairment | **3** Severe headache and severe CNS-impairment, e.g. power loss or dyskinesia (and somnolence > 50%); severe neurological impairment | **4** seizures or paralyses, unconsciousness (massive or life-threatening neurological impairment |
| **Peripheral nerves incl. spinal cord** | **0** normal | **1** mild L'Hermitte's syndrome, paraesthesiae | **2** marked L'Hermitte's syndrome with paraesthesiae and muscle weakness | **3** objective segmental neurological function loss, with paresis or paraesthesiae | **4** mono-, para- or quadraplegia |
| **Eyes** | **0** normal | **1** asymptomatic cataract, mild corneal ulceration or ceratitis (and / or conjunctivitis) | **2** Symptomatic cataract; moderate corneal ulceration (ceratitis); moderate retinopathy or glaucoma | **3** Severe ceratitis (ulceration), severe retinopathy with retinal detachment; severe glaucoma | **4** Massive ophthalmologic impairment; panophthalmitis; (uni-/ bilateral visual loss) |
| **Ears** | **0** normal | **1** mild external otitis with atrophy or fibrosis; no therapy; audiogram: hearing loss < 10 dB | **2** Moderate external otitis with atrophy or fibrosis; tinnitus; local therapy; moderate hearing loss (10 - 15 dB) | **3** Severe external et medial otitis; (continuous tinnitus not due to medication); severe hearing loss (> 15 - 20 dB) | **4** Severe osteochondritis, ulceration, necrosis; complete hearing loss on one or both sides (> 20 dB) |

**Adapted from:** Cox JD, Stetz J, Pajak TF. Toxicity criteria of the Radiation Therapy Oncology Group (RTOG) and the European Organization for Research and Treatment of Cancer (EORTC). Int J Radiat Oncol Biol Phys 1995;31:1341-1346.

**Comments:**...........................................................................................................................................

**Growth/Puberty:**

**Heigth in cm:**____________ **Height if seated in cm:** ____________

Weight in kg: ____________

Scoliosis: ☐ no ☐ yes

Deformation of bones or soft tissue: ☐ no ☐ yes, localization:...........................................

Description: .......................................................................................................................

.......................................................................................................................................

.......................................................................................................................................

Puberty development (after irradiation of brain, pelvis or upper part of lower extremity):

**Girls** :Menarche: ☐ no ☐ yes

Tanner stage breast:.......................................

Tanner stage hair ....................

Blood values: LH: ...............

FSH: ...............

Estradiol: ..............

Prolaktin: ..............

**Boys**: Tanner stage male genitals:...................

Tanner stage hair:......................

Volume testes: ..............

Blood values: LH: ...............

FSH: ...............

Testosterone: ...............

Prolaktin: ...............

**Blood values of the thyroid gland (after RT of head/neck/mediastinum):**

TSH basal: __________µE / ml

fT4: __________ng / dl

fT3: __________ng/dl

Date:_________________

**Current medication for thyroid gland:**

Name:______________________

Dosage:_____________________

**After RT of CNS:**

neurocognitive test performed: ☐ no ☐ yes

pathological result: ☐ no ☐ yes

.......................................
Name of Physician

......................................................
Signature / Stamp of Department.
