# Supplementary material for: Study protocol of the German "Registry for the Detection of Late Sequelae after Radiotherapy in Childhood and Adolescence" (RiSK)
Source: Radiat Oncol. 2008 Apr 21;3:10. doi: 10.1186/1748-717X-3-10 (PMC2373793; doi:10.1186/1748-717X-3-10)
Supplement: Additional file 2 — RiSK-Dokumentationsbogen Deutsch. This file is the original German version of the RiSK documentation form. [file 1748-717X-3-10-S2.pdf]

RiSK

RADIOTHERAPIE – BASISDOKUMENTATION

-

Primärtherapie: ☐ Rezidivtherapie : ☐

Name, Vorname : ....

Geburtsdatum:_____._____._________ Geschlecht : ☐ männlich ☐ weiblich

Straße:...; PLZ, Wohnort:…....................................

Diagnose : ....

Therapiestudie / Behandlungsprotokoll : ....

Behandelnde Kinderonkologie (Anschrift/Tel.) :..
....
....
....

Behandelnde Radioonkologie (Anschrift/Tel.,ggf. Stempel) : ....
....
....
....

Tumorsitz : ....

Zielgebiet der Bestrahlung: ....
(siehe Liste Seite 5)

Präradiotherapeutische Funktionseinschränkung im Bestrahlungsgebiet : ☐ nein

☐ ja, welche...

....

Präradiotherapeutische Funktionseinschränkung außerhalb des Bestrahlungsgebietes :☐ nein

☐ ja, welche:...

....

Körperhöhe vor Radiatio-Beginn in cm:_______ Sitzhöhe in cm (bei kraniospinaler Bestrahlung):__________ _

Körpergewicht vor Radiatio in kg:______________

Bögen zurücksenden an: RiSK-Studienzentrale, Klinik für Strahlentherapie – Radioonkologie -, Universitätsklinikum Münster, Albert-Schweitzer-Str. 33, 48129 Münster, Tel: +49-251/83-47387, Fax: +49-251-83-47355, Email: radtox@uni-muenster.de

Bestrahlungsplanung (bei 2 Tumorlokalisationen bitte eine 2. Tabelle verwenden)

	**Zielvolumen I**	**Zielvolumen II (Feldeinschränkung, Boost)**	**Zielvolumen III (Feldeinschränkung, Boost)**
Bestrahlter Organbereich:			
	☐Stehfeld<br>☐ Gegenfelder<br>☐> 2 Felder	☐Stehfeld<br>☐ Gegenfelder<br>☐> 2 Felder	☐Stehfeld<br>☐ Gegenfelder<br>☐> 2 Felder
Zahl der Bestrahlungsfelder :			
Nonkoplanare Felder :	☐ ja ☐ nein	☐ ja ☐ nein	☐ ja ☐ nein
Koplanare Felder :	☐ ja ☐ nein	☐ ja ☐ nein	☐ ja ☐ nein
Isodosenplan :	☐ ja ☐ nein	☐ ja ☐ nein	☐ ja ☐ nein
CT/MRT – gestützt :	☐ ja ☐ nein	☐ ja ☐ nein	☐ ja ☐ nein
3 D Planung :	☐ ja ☐ nein	☐ ja ☐ nein	☐ ja ☐ nein
Dosisvolumenhistogramm:	☐ ja ☐ nein	☐ ja ☐ nein	☐ ja ☐ nein
ICRU 50	☐ ja ☐ nein	☐ ja ☐ nein	☐ ja ☐ nein
Umschließende Isodose	%	%	%
½ Durchmesser	☐ ja ☐ nein	☐ ja ☐ nein	☐ ja ☐ nein
Herdtiefe	cm	cm	cm
Sonstige Dosierung			
Simulation	☐ ja ☐ nein	☐ ja ☐ nein	☐ ja ☐ nein
Kobalt 60	☐ ja ☐ nein	☐ ja ☐ nein	☐ ja ☐ nein
Linac- Photonen	☐ ja ☐ nein	☐ ja ☐ nein	☐ ja ☐ nein
Linac-Elektronen:<br>Energie:	☐ ja ☐ nein<br>____ MeV	☐ ja ☐ nein<br>____ MeV	☐ ja ☐ nein<br>____MeV
Neutronen	☐ ja ☐ nein	☐ ja ☐ nein	☐ ja ☐ nein
Protonen	☐ ja ☐ nein	☐ ja ☐ nein	☐ ja ☐ nein
Mischtechnik	☐ ja ☐ nein	☐ ja ☐ nein	☐ ja ☐ nein
Bolusmaterial	☐ ja ☐ nein	☐ ja ☐ nein	☐ ja ☐ nein
Stereotakt. Bestrahlung	☐ ja ☐ nein	☐ ja ☐ nein	☐ ja ☐ nein

Bestrahlungstechnik

☐ Brachytherapie:

☐endoluminal ☐ interstitiell ☐ Flab-Technik ☐ Seed-Implantation

☐ Intraoperative Bestrahlung

☐ Elektronen ☐Brachytherapie (Flab)

☐ Offene Radionuklide

☐ Lagerungshilfe (welche?)   ☐ Gesichtsmaske   ☐ Vakuumkissen   ☐Gipsschale

☐ Sedierung/Kurznarkose   ☐ Stereotaxiering

☐ andere...................……………………......................

Bestrahlungsplanung (bei 2 Tumorlokalisationen bitte eine 2. Tabelle verwenden)

	**Zielvolumen I**	**Zielvolumen II**	**Zielvolumen III**
Bestrahlungsbeginn :			
Bestrahlungsende :			
Anzahl der Bestrahlungstage :			
Bestrahlungspausen :	☐ ja , ☐☐ Tage<br>☐ nein	☐ ja , ☐☐ Tage<br>☐ nein	☐ ja , ☐☐ Tage<br>☐ nein
Grund :			
Gesamtdosis im Zielvolumen(Gy) :			
Einzeldosis (Gy) :			
Fraktionierung:	.... x.......... Gy /Woche	.... x.......... Gy /Woche	.... x.......... Gy /Woche

Bemerkungen:..

☐ Ganzkörperbestrahlung

Datum der Behandlung:...............................

Einzeldosis (Gy) :			Lungendosis :	
Fraktionierung:	.... x.......... Gy /Tag		Leberdosis	
Ganzkörperdosis (gesamt)			Nierendosis :	
Dosisleistung :				

<u>Bereits früher Strahlentherapie erfolgt?</u> ☐ nein ☐ja

falls ja, Zielgebiet:......................................   Dosis:...................................Gy

Bestrahlungsjahr:..........................

Operation im Bestrahlungsfeld : ☐ ja ☐ nein ☐ nicht bekannt

vor Bestrahlung erfolgt : ☐ ja ☐ nein

nach Bestrahlung vorgesehen: ☐ ja ☐ nein

Art der Operation:..

Chemotherapie vor Radiotherapie : ☐ ja ☐ nein ☐ nicht bekannt

Substanzen:.. ....

....

Chemotherapie simultan zur Radiotherapie: ☐ ja ☐ nein ☐ nicht bekannt

Substanzen:.. ....

....

Chemotherapie nach Radiotherapie : ☐ ja ☐ nein ☐ nicht bekannt

Substanzen:..

....

☐ Therapieassoziierte Akut-Toxizität EORTC / RTOG Grad 3 oder 4 während der Strahlentherapie

a)..

b)..

c)..

....

Datum Name des Arztes Unterschrift/Klinikstempel

Zielgebiet der Bestrahlung

1	Haut			**Abdomen**
2	Ganzkörper		42	Abdomen
3	Halbkörper		43	Leber
	**ZNS**		44	Leberhilus
81	kraniospinale Achse		45	Milz, Milzstiel
4	Hirnschädel		46	Magen
5	Großhirn		47	Paraaortale LK
6	Kleinhirn		48	Pankreas
7	Hirnstamm		49	Niere
8	Hypophyse		50	Nebenniere
9	Pinealisloge		51	Iliakale LK
10	Suprasellär		52	Inguinale LK
11	Kalotte		53	Kleines Becken
12	Siebbein		54	Beckenwand
13	Orbita		55	Rectum
14	Retrobulbär		56	Sakralhöhle
	**Kopf-Hals**		57	Anal
15	Wange		58	Blase
16	Waldeyerscher Rachenring		59	Prostata
17	Tonsille		60	Hoden
18	Schilddrüse		61	Uterus
19	Epipharynx		62	Parametrien
20	Mesopharynx		63	Ovar
21	Hypophaynx		64	Vagina
22	Oberkiefer		65	Vulva
23	Unterkiefer			**Myelon + Wirbelkörper**
24	Kieferhöhle		66	Myelon
25	Zunge		67	HWS
26	Mundboden		68	BWS
27	Larynx		69	LWS
28	Submentale LK		70	Sacrum
29	Cervikale LK		71	Paravertebral
30	Supra-/infraclavikuläre LK			**Extremitäten**
	**Thorax**		72	Schulter / Hals
31	Mediastinum		73	Oberarm
32	Hilomediastinal		74	Unterarm
33	Hilus		75	Hand
34	Oesophagus		76	Hüfte
35	Herz / Myocard		77	Oberschenkel
36	Lunge		78	Unterschenkel
37	Lunge zentral		79	Fuß
38	Lunge peripher			
39	Brustwand		80	Sonstige:
40	Mamma			
41	Axilla			

RiSK

RADIOTHERAPIE – DOKUMENTATION

- Organdosen -

Richtlinien zur Organdosendokumentation:

- **Im Folgenden werden lediglich die Bestrahlungsdosen von Organen oder Organbereichen im Strahlenfeld dokumentiert. Strahlenexposition außerhalb des Strahlenfeldes soll nur für die Ovarien / Hoden und die Schilddrüse angegeben werden.**

- **Bei Schilddrüse und Hoden ist die dosimetrische Erfassung vorgesehen.** Dabei sollten wenn möglich mehrere Messpunkte über das Organ verteilt gewählt werden. Bei einer Messung eines einzelnen Punktes sollte eine repräsentative Stelle in der Mitte des Organs gewählt werden.

- **Für Lunge, Herz, Leber und Nieren müssen die Dosen aus dem Dosisvolumenhistogramm angegeben werden**.

- **D-max: maximale Punktdosis im Organ**
  D-min: minimale Punktdosis im Organ

- **I. Teil: Dokumentation von Dosen an Knochen, Weichteilen, Gelenken**
  II. Teil: Dokumentation nach Organen, von kranial nach kaudal gegliedert.

I. Teil

KNOCHENMARK

Bitte Feldkontur anzeichnen

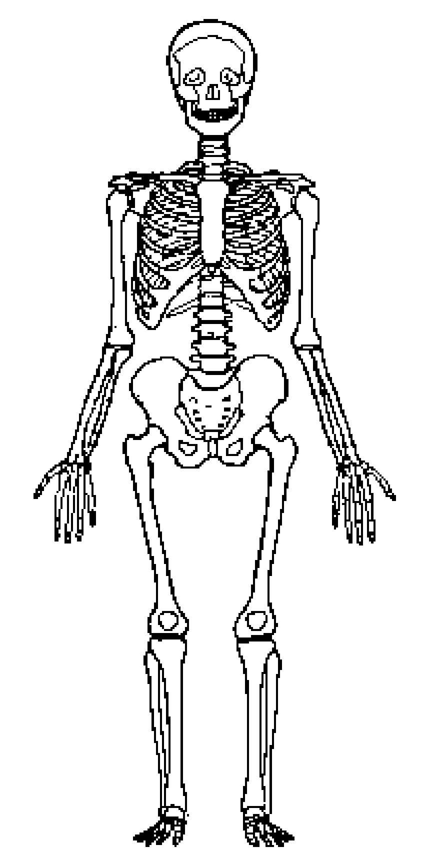

(Bestrahlungsbereich markieren)

WIRBELSÄULE UND RÜCKENMARK

Dosisdokumentation und Toxizitätsnachsorge für Wirbelkörper und Myelon ab 10 Gy

Angabe der bestrahlten Wirbelkörper: von...............................bis.................................

D-max in den bestrahlten WK: ....Gy D-min in den bestrahlten WK:............Gy

Dosisgradient unterhalb 20Gy im Wirbelkörper? ☐ ja ☐ nein

Angabe der bestrahlten Myelonabschnitte: von...............................bis................................

D-max im bestrahlten Segment: ....Gy D-min im bestrahlten Segment:............Gy

BEWEGUNGSAPPARAT

KNOCHEN UND WEICHTEILE

Dosisdokumentation und Toxizitätsnachsorge wenn mit ≥ 10 Gy bestrahlt

	D-max (Gy)	Gesamter Knochen im Zielvolumen
Kalotte		————
Rippen re.		————
Rippen li.		————
Becken re.		
Becken li.		
Sacrum		

	D-max (Gy)	Gesamter Knochen im ZV		proximale Epiphysenfuge im Feld			distale Epiphysenfuge im Feld		
		Ja	nein	ja	partiell	nein	Ja	partiell	nein
Oberarm re.									
Radius re.									
Ulna re.									
Hand re.				——	——	——	——	——	——
Oberschenkel re.									
Tibia re.									
Fibula re.									
Fuß re.				——	——	——	——	——	——
Oberarm li.									
Radius li.									
Ulna li.									
Hand li.				——	——	——	——	——	——
Oberschenkel li.									
Tibia li.									
Fibula li									
Fuß li.				——	——	——	——	——	——

Gesamte Extremitäten-Zirkumferenz im Feld : ☐ ja (ohne Lymphstreifen) ☐ nein

GELENKE

Dosisdokumentation und Toxizitätsnachsorge für Gelenke wenn mit ≥ 20 Gy bestrahlt

Gelenke im Bestrahlungsfeld: ☐ nein ☐ ja

	Rechts (Gy)	Links (Gy)
Schultergelenk		
Ellenbogengelenk		
Handgelenk (radio-carpal)		
Hüftgelenk		
Kniegelenk		
Sprunggelenk		

II. Teil (Organdosen)

ZNS

Dosisdokumentation und Toxizitätsnachsorge, wenn im Zielvolumen

☐ **Helmfeld bei ZNS Prophylaxe / ZNS-Befall bei ALL / AML**

verordnete Dosis:..............Gy

Dosimetrie Schilddrüse: ermittelte Gesamtdosis:......../……../……../……../……..Gy

(oder Dosisangabe aus Plan), alternativ: Abstand der Schilddrüse vom Feldrand:………..cm

(**keine weitere Dokumentation nötig**)

☐ **Ganzhirn-Bestrahlung,** verordnete Dosis:............ Gy

☐ **kranio-spinale Bestrahlung,** verordnete Dosis:............ Gy

Boost spinal: ☐ nein ☐ ja: kumulative Dosis:..........Gy
Myelonabschnitt:.…..................

Aufsättigung hintere Schädelgrube:………..Gy

Boost cerebral: ☐ nein ☐ ja: kumulative Dosis:..........Gy
Region:............................

☐ **Teilhirn-Bestrahlung**

☐ < 20 Gy → keine Dokumentation notwendig

☐ > 20 Gy

	D-max (Gy)	D-min (Gy)
Hypophyse		
Chiasma opticum		
Hirnstamm		

Rückenmark: siehe Teil I

Orbita

Dosisdokumentation und Toxizitätsnachsorge bei jeder eingestrahlten Dosis.

	D-max (Gy)	Gesamtes Organ im ZV	
		ja	nein
Auge re.			
Linse re.		————	
Tränendrüse re.			
Nervus opticus re.			

	D-max (Gy)	Gesamtes Organ im ZV	
		ja	nein
Auge li.			
Linse li.		————	
Tränendrüse li.			
Nervus opticus li.			

OHR

Dosisdokumentation und Toxizitätsnachsorge bei jeder eingestrahlten Dosis.

	D-max (Gy)
Mittelohr re.	
Innenohr re.	

	D-max (Gy)
Mittelohr li.	
Innenohr li.	

Parallele Chemotherapie mit Cisplatin:
☐ ja ☐ nein

SPEICHELDRÜSEN

Dosisdokumentation und Toxizitätsnachsorge , wenn mit ≥ 20 Gy bestrahlt.

	D-max (Gy)	Komplett im Feld ?	
Gl. parotis re.		☐ja	☐nein
Gl. submandibularis re.		☐ja	☐nein
Gl. parotis li.		☐ja	☐nein
Gl. submandibularis li.		☐ja	☐nein

ZÄHNE / KIEFER

Dosisdokumentation und Toxizitätsnachsorge bei jeder eingestrahlten Dosis.

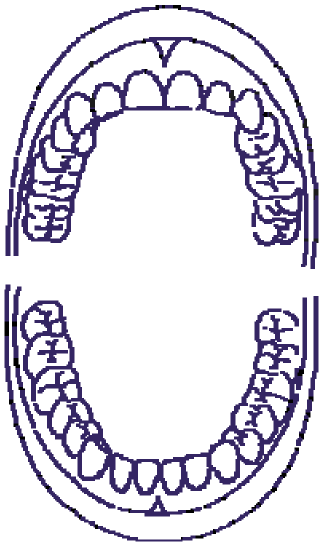

Bitte den bestrahlten Bereich markieren

Kiefergelenk links im Bestrahlungsfeld ☐ nein ☐ ja, Dosis:..................Gy

Kiefergelenk rechts im Bestrahlungsfeld ☐ nein ☐ ja Dosis:..................Gy

SCHILDDRÜSE

Dosisdokumentation und Toxizitätsnachsorge bei jeder eingestrahlten Dosis.
<u>Dosimetrische Erfassung</u> von Streustrahlung z. B. bei Schädel- oder Mediastinalbestrahlung; alternativ Abstandsangabe (cm) zum Feldrand.

☐ **Schilddrüse im Zielvolumen** D-max:...................Gy

Gesamtes Organ im Zielvolumen: ☐ Nein ☐ Ja

☐ **Schilddrüse nicht im Zielvolumen**
(bei Kopf-Hals- oder Mediastinal-Bestrahlung / bei kraniospinaler Achsenbestrahlung)
(mehrere Meßpunkte)

Dosimetrie: ☐ ja → ermittelte Gesamtdosis:……../……../……../……../……..Gy
(oder Dosisangabe aus Plan)
☐ nein→ Abstand zum Feldrand:...................cm

KEHLKOPF

Dosisdokumentation und Toxizitätsnachsorge bei Bestrahlung ≥ 20 Gy.

D-max: ....Gy

Ausmaß des bestrahlten Organanteils ≥ 20 Gy: ☐ < 1/3 ☐ 1/3 – 2/3 ☐ > 2/3 ☐ komplett

MAMMA

Dosisdokumentation und Toxizitätsnachsorge bei jeder eingestrahlten Dosis.

	D-max (Gy)	Gesamte Mamma im ZV	
		Ja	Nein
Mamma re.			
Mamma li.			

Symmetrische Bestrahlung beider Mammae : ☐ ja ☐ nein

ÖSOPHAGUS

Dosisdokumentation und Toxizitätsnachsorge bei Teildosis ≥ 40 Gy. Abschätzung der bestrahlten Länge anhand der Simulationsaufnahmen.

Bestrahlter Ösophagusabschnitt ≥ 40 Gy

von........................... bis .... (Angabe der Wirbelsegmente)

D-max im bestrahlten Segment: .... Gy

BAUCHSPEICHELDRÜSE

Dosisdokumentation und Toxizitätsnachsorge bei (Teil)Bestrahlung ≥ 20 Gy (Volumenabschätzung nach Simulation/Plan)

D-max:...................Gy

Ausmaß des bestrahlten Organanteils ≥ 20 Gy:
☐ < 1/3
☐ 1/3 – 2/3
☐ > 2/3
☐ komplett

HARNBLASE

Dosisdokumentation und Toxizitätsnachsorge wenn im Zielvolumen.

D-max:...................Gy; Harnblase komplett im ZV: ☐ nein ☐ ja

DOSISVOLUMENHISTOGRAMM

HERZ

Dosisdokumentation und Toxizitätsnachsorge bei einer Dosis ≥ 15 Gy. Erfassung auch bei infradiaphragmaler Bestrahlung. Dosisvolumenhistogramm!.

LUNGE

Dosisdokumentation und Toxizitätsnachsorge bei einer Ganzlungendosis ≥ 10 Gy bzw. Teillungendosis ≥ 18 Gy. Dosisvolumenhistogramm!

LEBER

Dosisdokumentation und Toxizitätsnachsorge bei Bestrahlung vom ≥ 1/3 der Leber und/oder Teilleberbestrahlung ≥ 20 Gy. Dosisvolumenhistogramm!

NIEREN

Bestrahlungsdokumentation und Toxizitätsnachsorge, wenn ≥ 1/2 einer Niere bestrahlt ist und/oder eine Teilorgandosis von ≥ 12 Gy vorliegt. Dosisvolumenhistogramm!

Alternativ: DVH in Kopie oder Eintrag in die Tabelle

Dosis	Herz	Lunge li.	Lunge re.	Lunge gesamt	Leber	Niere li.	Niere re.
> 5 Gy	%	%	%	%	%	%	%
>10 Gy	%	%	%	%	%	%	%
>15 Gy	%	%	%	%	%	%	%
>20 Gy	%	%	%	%	%	%	%
>30 Gy	%	%	%	%	%	%	%
>40 Gy	%	%	%	%	%	%	%
>50 Gy	%	%	%	%	%	%	%
>60 Gy	%	%	%	%	%	%	%

Vorbestehende Funktionseinschränkungen an Herz, Lunge, Leber, Niere:

☐ nein ☐ ja,

welche...

....

DÜNN- UND DICKDARM

Dosisdokumentation und Toxizitätsnachsorge bei (Teil)Abdomenbestrahlung ≥ 10 Gy. (Volumenabschätzung nach Simulation/Plan)

Dünndarm: D-max:..................Gy

Ausmaß des bestrahlten Organanteils ≥ 20 Gy:

☐ < 1/3

☐ 1/3 – 2/3

☐ > 2/3

☐ komplett

Dickdarm: D-max:...............Gy

☐ < 1/3

☐ 1/3 – 2/3

☐ > 2/3

☐ komplett

..

VAGINA / UTERUS

Dosisdokumentation und Toxizitätsnachsorge wenn im Zielvolumen.

	Dosis im ZV
Vagina	
Cervix	
Uterus	

Vaginaanteil im behandelten Volumen:

☐ Teilbestrahlung (Angabe bei kleinen Kindern evtl. nicht möglich)

☐ komplett

OVARIEN

Dosisdokumentation und Toxizitätsnachsorge bei jeder eingestrahlten Dosis

(sofern bestimmbar). Streustrahlanteil, wenn Feldrand ≤ 3cm vom Ovar entfernt.

Ovar rechts: ☐ im Bestr.-Feld, Dosis: → ....Gy
☐ Streustrahldosis: → ....Gy (Dosisangabe nach Bestrahlungsplan)

Ovar links: ☐ im Bestrahlungsfeld: → ....Gy
☐ Streustrahldosis: → .... Gy (Dosisangabe nach Bestrahlungsplan)

Ovarialverlagerung : ☐ nein ☐ ja →wohin verlagert: ....
....

HODEN

Dosisdokumentation und Toxizitätsnachsorge bei jeder eingestrahlten Abdomendosis. Dosimetrische Erfassung der Streustrahlung bei Bestrahlung Becken/Leisten/ Oberschenkel.

Bestrahlung mit Hodenkapsel? ☐ ja ☐ nein

Hoden rechts:
☐ im Bestrahlungsfeld → ....Gy
☐ Streustrahldosis: Dosimetrie
☐ ja→ermittelte Gesamtdosis (mehrere Messpunkte)
.........../........../........../........../...........Gy
☐ nein→ Abstand vom Feldrand in cm:.........

Hoden links:
☐ im Bestrahlungsfeld→ ....Gy
☐ Streustrahldosis: Dosimetrie
☐ ja→ermittelte Gesamtdosis:
........../........../........../...........Gy
☐ nein→Abstand vom Feldrand

....
Datum: **ausfüllender Strahlentherapeut** (Bitte Druckbuchstaben) **Unterschrift/Klinikstempel** (Bitte Telefonnummer)

Nr. Datum . .	Max. akute Nebenwirkungen nach Radiotherapie bei Kindern (RTOG/EORTC)	Pat.Name, Geb.-Dat.

**Allgemein-zustand, Karnofsky**	**0** normal<br>KI: 100%	**1** Gering geminderte Aktivität; voll ambulant,<br>KI: 70–80%	**2** Deutlich geminderte Leistungskraft; tags > 50% ambulant, Aktivität deutl. eingeschränkt.<br>KI: 50–60%	**3** Stark geminderte Leistungskraft; tags> 50% bettlägerig; pflegebedürftig<br>KI: 30–40%	**4** Lebensbedrohlicher Zustand; ständig bettlägerig und voll pflegebedürftig;<br>KI: < 30%
**Knochenmark**<br>Leukozyten (N/µl)<br>Neutrophile (N/µl)<br>Thrombozyten (*10^3/µl)<br>Hämoglobin (g/dl)	**0**<br>≥ 4.000<br>≥ 2.000<br>≥ 100<br>Altersnorm	**1**<br>3.000 - < 4.000<br>< 2.000 - 1.500<br>75 - < 100<br>10,0 - < N	**2**<br>2.000 - < 3.000<br>< 1.500 - 1.000<br>50 - < 75<br>8,0 - < 10,0	**3**<br>1.0 - < 2.000<br>< 1.000 - 500<br>10,0 - < 50<br>6,5 - < 8,0	**4**<br>< 1.000<br><500 / Sepsis<br>< 10,0<br>< 6,5
**Haut / Unterhaut**	**0** normal	**1** Geringes, blasses Erythem; Haarausfall; trockene Desquamation, reduzierte Schweißsekretion.	**2** Mäßiges Erythem, vereinzelt feuchte Epitheiolyse (≤ 50%), mäßiges Ödem; (lokale Therapie nötig)	**3** (Ausgeprägtes Erythem), konfluierende feuchte Epitheliolyse (> 50%), starkes Ödem;<br>(intensive Therapie nötig)	**4** Ulzeration, Nekrose, Blutung<br>(operative Therapie nötig)
**Schleimhäute**	**0** normal	**1** Geringes Enanthem oder Beläge, evtl. geringe Schmerzen, keine Analgetika nötig.	**2** (Mäßig) schmerzhafte, fleckförmige Mukositis mit entzündlichen serosanguinösen Belägen, milde Analgetika	**3** Konfluierend fibrinöse Mukositis; evtl. starke Schmerzen, die zentral wirksame Analgetika nötig machen	**4** Ulzeration, Blutung oder Nekrose;<br>(PEG / parenterale Ernährung)
**Speicheldrüsen**	**0** normal	**1** Geringe Mundtrockenheit; zäher Speichel; geringe Geschmacksstörung, z.B. metallisch; normale Kostform (etwas mehr Flüssigkeit)	**2** Mäßige bis nahezu vollständige Mundtrockenheit, sehr zäher Speichel; mäßige Geschmacksstörung; (viel Flüssigkeit, feste bis breiige Nahrung)	**3** Komplette Mundtrockenheit; kompletter Geschmacksverlust; keine festen Speisen mehr, flüssige Nahrung	**4** Akute Nekrose oder Ulzeration im Bereich der Speicheldrüsen.<br>(PEG / parenterale Ernährung)
**Pharynx/ Ösophagus**	**0** normal	**1** Geringe Dysphagie oder Odynophagie; evtl. lokale Anästhetika oder milde Analgetika; evtl. breiige Nahrung	**2** Mäßige Dysphagie oder Odynophagie; evtl. (starke Anästhetika und) zentral wirksame Analgetika; evtl. breiige oder flüssige Nahrung	**3** Ausgeprägte Dysphagie oder Odynophagie mit Dehydratation oder Gewichtsverlust (>15% vom Ausgangsgewicht), NG / PEG-Sonde nötig oder i.v. Lösungen oder Hyperalimentation	**4** Komplette Obstruktion; (tiefe) Ulzeration, Perforation oder Fistel; (PEG / parenterale Ernährung)
**Kehlkopf**	**0**	**1** Geringe intermittierende Heiserkeit / Reizhusten; geringes Schleimhauterythem, Stimmbandödem;<br>(keine Therapie)	**2** Ständige Heiserkeit, Sprechen möglich, Hals- und Ohrenschmerzen, fleckiges fibrinöses Exsudat oder mäßiges Stimmbandödem, keine Analgetika nötig; (mäßiger) Husten, Antitussiva nötig	**3** Flüstersprache; starke Hals- oder Ohrenschmerzen, zentrale Analgetika nötig; konfluierend fibrinöses Exsudat, ausgeprägtes Stimmbandödem	**4** Massive Dyspnoe, Stridor oder Hämoptysen, Intubation oder Tracheostomie erforderlich.
**Lunge**	**0** normale Blutgase:<br>pO_2: > 85;<br>pCO_2 < 40	**1** Geringe Symptomatik oder trockener Reizhusten oder geringe Belastungsdyspnoe<br>pO_2:71-85; pCO_2: 41-50	**2** Mäßiger, permanenter Reizhusten, zentrale Antitussiva nötig; deutliche Belastungs-, aber keine Ruhedyspnoe<br>pO_2:61-70; pCO_2: 51-60	**3** Ausgeprägter auf zentrale Antitussiva refraktärer Husten; starke Ruhedyspnoe; klinisch oder radiologisch akute interstinelle Pneumonitis<br>pO_2:51-60; pCO_2: 61-70<br>(immer wieder O_2- und Steroide nötig)	**4** Massive respiratorische Insuffizienz; pO_2 < 50; pCO_2 > 70)<br>ständige O2-Gabe oder kontrollierte Beatmung nötig
**Herz**					
**Arryhthmie**	**0** keine	**1** Asympt., keine Therapie	**2** rekurr. / persist., keine Therapie	**3** Therapie erforderlich	**4** (Hypotension, ventr. Arrhth., Defibrillation)
**Herzfunktion**	**0** normal	**1**Asymptomat. EF ↓ (Ruhe) > = 10 % aber < 20 % vom Ausgangswert	**2** Asymptomat., aber EF ↓ (Ruhe) unter dem unteren Normwert für EF (Arbeit) oder EF ↓ > = 20 % vom Ausgangswert	**3** Milde CHF, therapeutisch kompensiert	**4** schwere / refraktäre CHF oder Notwendigkeit der Intubation
**Echocardio: LV-SF %**	**0** > = 30	**1** > = 24 - < 30	**2** > = 20 - < 24	**3** > 15 - < 20	**4** < = 15
**Oberer GI-Trakt, Magen und Dünndarm**	**0** normal	**1** Geringe Anorexie (≤ 5% Gewichtsverlust), Übelkeit;(einmaliges Erbrechen); geringe abdominelle Schmerzen; keine spezifische Therapie (Parasympatolytika oder Analgetika)	**2** Mäßige Anorexie (≤15% Gewichtsverlust), Übelkeit oder Erbrechen (2 bis 5 mal), Antiemetika nötig, mäßige Bauchschmerzen: leichte Parasympatholytika oder Analgetika nötig)	**3** Ausgeprägte Anorexie (> 15% Gewichtsverlust); Übelkeit oder Erbrechen (6 bis 10 mal); NG / PEG-Sonde oder parenterale Ernährung nötig; ausgeprägte Bauchschmerzen trotz Medikation; Hämatemesis, Mälena; starke Blähungen (Rö:weitgestellte Darmschl.)	**4** Ileus; (sub) akute Darmobstruktion Fistel, Perforation; GI-Blutung, Transfusionen nötig; massivste Bauchkrämpfe, die operative Druckentlastung oder Umgehungsoperation nötig machen

**Unterer GI-Trakt; Dick- und Enddarm mit Beckenregion**	**0** normal	**1** Öfter Stuhlgang oder veränderte (reduzierte) Stuhlkonsistenz, keine Medikamente nötig; (geringe) rektale Beschwerden, keine Analgetika nötig.	**2** (Mäßige) Diarrhoe Parasympatholytika nötig (z.B. Diphenoxylat); (häufig) Schleimabgänge, aber keine Einlagen nötig; rektale oder abdominale Tenesmen, milde Analgetika nötig.	**3** (Ausgeprägte) Diarrhoe die parenterale Ernährung nötig macht. Ausgeprägte Blut- und Schleimabgänge, Einlagen nötig; starke Blähungen (Röntgen zeigt weite Darmschlingen); (zentral wirksame Analgetika)	**4** (Sub) akute Darmobstruktion (Ileus), Darmfistel oder Perforation; Darmblutung, Transfusion nötig; schwerste abdominale Schmerzen, operative Entlastung oder Umleitung
**Leber**	**0** normal	**1** Geringe Müdigkeit, Übelkeit, Dyspepsie,	**2** Mäßige Symptomatik (Müdigkeit, Übelkeit, Dyspepsie)	**3** Ausgeprägte Leberinsuffizienz	**4** Lebernekrose, hepatogene(s) Koma oder Enzephalopathie; (parenterale Ernährung, portocavaler Shunt erforderlich)
**Bilirubin**	**0** Altersnorm (N)	**1** > N – 1,5 x N	**2** > 1,5 – 3,0 x N	**3** > 3,0 – 10,0 x N	**4** > 10,0 x N
**S-GOT / S-GPT**	**0** Altersnorm (N)	**1**> N – 2,5 x N	**2** > 2,5 – 5,0 x N	**3** > 5,0 – 20,0 x N	**4** > 20,0 x N
**Urogenitaltrakt (und Niere)**	**0** normal	**1** Wasserlassen / Nykturie 2 mal häufiger als sonst; (geringe) Dysurie und Harndrang; keine Medikamente	**2** Wasserlassen oder Nykturie viel häufiger als sonst, <1 mal/ h; (mäßige) Dysurie, Harndrang und Blasenkrämpfe, lokale Anästhetika nötig (z.B.Phenazopyridin)	**3** Wasserlassen oder Nykturie sehr häufig, >1 mal/ h; ausgeprägte) Dysurie, Beckenschmerzen und Blasenkrämpfe, regelmäßig zentral wirksame Analgetika(Narkotika) nötig; ausgeprägte Hämaturie mit oder ohne Blutkoagel	**4** (Lebensbedrohliche) Hämaturie, Bluttransfusionen nötig, akute Blasenobstruktion, nicht infolge Hämaturie oder Blutkoageln, Ulzeration oder Nekrose
**Kreatinin (mg%)**<br>**Kreatinin-Clearence**<br>**Proteinurie (g/l)**<br>**Hämaturie**	**0** Altersnorm (N)<br>> 90<br>keine<br>keine	**1** > N – 1,5 x N<br>60 – 89<br>< 3<br>mikroskopisch	**2** > 1,5 – 3,0 x N<br>40 –59<br>3 – 10,0<br>makroskopisch ohne Koageln	**3** > 3,0 – 6,0 x N<br>20 – 39<br>> 10,0<br>makroskopisch mit Koageln	**4** > 6,0 x N<br>< 19<br>Nephrotisches Syndrom<br>Transfusion erforderlich
**Harnleiter und Blase**	**0** normal	**1** Geringe Schleimhautatrophie; geringe (kleinflächige) Teleangiektasie; (Harndrang gering verstärkt); Mikrohämaturie	**2** Mäßig verstärkter Harndrang; generalisierte Teleangiektasie; zeitweilig Makrohämaturie; (Polyurie: > 6mal/Tag oder < einmal pro Stunde)	**3** Massiver Harndrang (>einmal pro Stunde) und Dysurie; ausgeprägte generalisierte Teleangiektasie (oft mit Petechien); häufige Hämaturie	**4** Nekrose, (Perforation, Fistel) schwere Blasenkontraktur
**Knochen**	**0** normal	**1** Keine Symptome; keine Wachstumsverzögerung; (gering) reduzierte Knochendichte	**2** Mäßige Schmerzen oder Spannungsgefühl; (mäßige) Wachstumsstörung; irreguläre Knochensklerosierung	**3** Ausgeprägte Schmerzen oder Spannungsgefühl; völliger Wachstumsstillstand, dichte Knochensklerosierung	**4** Nekrose bzw. Osteoradionekrose; Spontanfraktur
**Gelenke**	**0** normal	**1** Geringe Gelenksteife; (keine Symptome) geringe Funktionseinbußen	**2** Mäßige Gelenksteife; zeitweilige oder mäßige Gelenkschmerzen; mäßige Funktionseinbußen	**3** Ausgeprägte Gelenksteife; ausgeprägte Gelenkschmerzen mit ausgeprägten Funktionseinbußen	**4** Nekrose, vollständige Gelenksteife (Ankylose mit Funktionsverlust)
**Zentrales Nervensystem (Gehirn)**	**0** normal	**1** Voller funktioneller Status (Arbeitsfähigkeit) mit geringen neurologischen Ausfällen; keine spezifische Therapie nötig	**2** (Mäßige) neurologische Ausfälle; häusliche Pflege, pflegerische Hilfe nötig; Medikation inkl. (niedrig dosiert) Steroide; evtl. Antikonvulsiva	**3** (Ausgeprägte) neurologische Ausfälle, Krankenhauseinweisung für erste Therapiemaßnahmen nötig; (hochdosiert Steroide oder Antikonvulsiva)	**4** Lebensbedrohliche neurologische Ausfälle (Paralyse, Koma), > 3 Krampfanfälle pro Woche trotz Medikation; Hospitalisierung
**Peripheres Nervensystem (Rückenmark)**	**0** normal	**1** Diskretes L'hermitte-Zeichen (Parästhesien; reduzierte Reflexe)	**2** Ausgeprägte L'hermitte-Zeichen (mit Parästhesien oder Muskelschwäche)	**3** Objektive (segmentale) neurologische Ausfälle (mit Parästhesien oder Paresen)	**4** Mono-, Para-Teraplegie
**Augen**	**0** normal	**1** geringe Konjunktivitis mit oder ohne Sklerainjektion; verstärktes Augentränen, keine Therapie	**2** Mäßige Konjunktivitis mit oder ohne Keratitis, Steroide oder Antibiotika nötig; „trockene Augen", künstliche Tränenflüssigkeit nötig; Iritis mit dadurch bedingter Photophobie	**3** Ausgeprägte Kreatitis mit Ulzeration der Kornea; objektiver Visusverlust bei Sehgenauigkeits-oder Gesichtsfeldanalyse; akutes Glaukom; Panophtalmitis	**4** (Massive ophathalmologische Komplikationen und Störungen) uni- oder bilaterale Erblindung
**Ohren**	**0** normal	**1** Geringe Otitis externa mit Erythem und Juckreiz infolge von trockener Hautschuppung, keine Therapie; Audiometrie unverändert gegenüber der Ausgangslage (oder: <10 dB)	**2** Mäßiggerade (seröse) Otitis externa et media, lokale Medikamente nötig; nur im Test mäßige Hypakusis (oder: 10-15 dB); (zeitweilig Tinnitus)	**3** Ausgeprägte (serisanguinöse) Otitis externa (et media); symptomatische Hypakusis (oder: >15-20 dB); (ständiger) Tinnitus, nicht medikamentös bedingt	**4** (Schwere Osteochondritis; Ulzeration, Nekrose) vollständige (ein- oder beidseitige) Ertaubung (oder :> 20 dB)

....
Ausfüllender Arzt

....
Unterschrift/Klinikstempel

Nr.	Datum	Chronische Strahlenfolgen bei Kindern (RTOG/EORTC)	Pat.Name, Geb.-Dat.
	. .		

Status	Komplette Remission	Progression	Zweit-Malignom	Verstorben	Todesdatum
**Allgemein-zustand**	**0** normale Aktivität, keine Beeinträchtigung	**1** geringe Beeinträchtigung der Aktivität, jedoch keine zusätzliche Hilfe erforderlich	**2** altersentsprechende Aktivität stark eingeschränkt (z.B. kein regelmäßiger Kindergarten- bzw. Schulbesuch möglich)	**3** Bettlägerig, pflegebedürftig	**4** intensive Behandlung notwendig, schwerstkrank, moribund
**Karnofsky**	**0** 100%	**1** 70–80%	**2** 50–60%	**3** 30–40%	**4** < 30%
**Hb** *(g/dl)*	**0** Altersnorm	**1** 10,0 - < N	**2** 8 – < 10,0	**3** 6,5 – < 8,0	**4** < 6,5
**Leuko** *(N/µl)*	**0** ≥ 4.000	**1** 3.000 – < 4.000	**2** 2.000 – < 3.000	**3** 1.000 – < 2.000	**4** < 1.000
**Thrombo** *(1000/µl)*	**0** ≥ 100	**1** 75 – < 100	**2** 50 – < 75	**3** 10 - < 50	**4** < 10
**Haut**	**0** normal	**1** geringe Atrophie, Pigmentveränderungen, geringer Haarverlust	**2** flächige Atrophie, mäßige Teleangiektasie (≤ 50%), völliger Haarverlust	**3** ausgeprägte Atrophie, ausgeprägte Teleangiektasie (> 50%)	**4** Ulzeration, Nekrose, (operative Therapie nötig)
**Unterhaut-gewebe**	**0** normal	**1** geringe asymptomatische Fibrose, ohne Kontraktur, gering reduziertes subkutanes Fettgewebe	**2** mäßige, asymptomatische Fibrose mit ≤ 10% linearer Kontraktur, mäßig reduziertes subkutanes Fettgewebe	**3** ausgeprägte (symptomatische) Fibrose mit >_10% linearer Kontraktur, stark reduziertes subkutanes Fettgewebe	**4** Ulzeration, Nekrose, (operative Therapie nötig)
**Schleimhäute**	**0** normal	**1** geringe Atrophie oder Trockenheit	**2** mäßige Atrophie und Teleangiektasie; reduzierte Schleimproduktion	**3** ausgeprägte Atrophie und Teleangiektasie, Verlust der Schleimproduktion	**4** Ulzeration, Nekrose, (operative Therapie nötig)
**Speicheldrüsen**	**0** normal	**1** geringe Mundtrockenheit aber gute Stimulierbarkeit (normale Kost/Ernährung)	**2** mäßige Mundtrockenheit, geringe Stimulierbarkeit, (viel Flüssigkeit, breiige Nahrung)	**3** komplette Mundtrockenheit, keine Stimulierbarkeit, (keine festen Speisen, flüssige Nahrung)	**4** Fibrose (komplette Atrophie) (PEG / parenterale Ernährung)
**Pharynx/ Ösophagus**	**0** normal	**1** geringe Fibrose, geringe Schluckstörungen bei festen Speisen, keine Schmerzen beim Schlucken (normale Kostform/Ernährung)	**2** (mäßige Fibrose), keine normale Aufnahme von festen Speisen, halbfeste (breiige) Nahrung, Dilatation ggf. nötig	**3** ausgeprägte Fibrose (oder Dysphagie), nur Aufnahme von Flüssigkeit möglich, Dilatation nötig, Schmerzen beim Schlucken möglich	**4** Nekrose, Perforation, Fistel (operativer Eingriff nötig oder PEG/ parenterale Ernährung)
**Larynx**	**0** normal	**1** (geringe) Heiserkeit (oder Reizhusten), geringes Stimmbandödem	**2** (mäßige Heiserkeit oder Reizhusten), mäßiges Stimmbandödem, Chondritis (symptomatische Therapie)	**3**(„Flüstersprache"), ausgeprägtes Stimmbandödem, massive Chondritis, (intensive Lokaltherapie, Analgetika)	**4** Nekrose, (massive Dyspnoe und Stridor, Ulzeration, Intubation oder Tracheotomie**)**
**Lunge**	**0** normal pO2: > 85; pCO2 < 40	**1** keine oder geringe Symptome (trockener Reizhusten); geringe radiologische Zeichen (geringe Belastungsdyspnoe) pO2:71-85; pCO2: 41-50	**2** mäßig symptomatische Lungenfibrose oder Pneumonitis (massiver Husten); geringes Fieber; radiologisch fleckige Veränderungen; (mäßige Belastungsdyspnoe) pO2:61-70; pCO2: 51-60	**3** Ausgeprägte symptomatische Lungenfibrose oder Pneumonitis; radiologisch dichte Veränderungen; (starke Ruhedyspnoe) pO2:51-60; pCO2: 61-70 (intensive medikamentöse Therapie)	**4** Massive respiratorische Insuffizienz; permanente O2-gabe und kontrollierte Beatmung nötig pO2 < 50; pCO2 > 70 (intensivmedizinsche Maßnahmen)
**Herz**					
**Arryhthmie**	**0** keine	**1** Asympt., keine Therapie	**2** rekurr. / persist. keine Therapie	**3** Therapie erforderlich	**4** (Hypotension, ventr. Arrhth., Defibrillation)
**Herzfunktion**	**0** normal	**1**Asymptomat. EF ↓ (Ruhe) > = 10 % aber < 20 % vom Ausgangswert	**2** Asymptomat. EF ↓ (Ruhe) unter dem unteren Normwert für EF (Arbeit) oder EF ↓ > = 20 % vom Ausgangswert	**3** Milde CHF, therapeutisch kompensiert	**4** schwere / refraktäre CHF oder Notwendigkeit der Intubation
**Echo: LV-SF %**	**0** > = 30	**1** > = 24 - < 30	**2** > = 20 - < 24	**3** > 15 - < 20	**4** < = 15
**Oberer GI-Trakt**	**0** normal	**1** Geringe Anorexie (≤ 5% Gewichtsverlust); Übelkeit; (einmal Erbrechen); geringe abdominelle Schmerzen : keine spezifische Therapie (Parasympatholytika oder) Analgetika)	**2** mäßige Anorexie (≤ 15% Gewichtsverlust); Übelkeit oder Erbrechen (2 bis 5 mal), Antiemetika nötig; mäßige Bauchschmerzen : leichte (Parasympatholytika oder) Analgetika nötig	**3** Ausgeprägte Anorexie (> 15% Gewichtsverlust); Übelkeit oder Erbrechen (6 bis 10mal); NG-/PEG-Sonde oder parenterale Ernährung nötig; ausgeprägte Bauchschmerzen trotz Medikation; Hämatemesis, Mälena; starke Blähungen	**4** Nekrose; Perforation, Fistel; komplette Obstruktion (Ileus); GI-Blutung; PEG oder parenterale Ernährung, Transfusionen, OP nötig

**Unterer GI-Trakt Dünn- und Dickdarm**	**0** normal	**1** Gering reduzierte Stuhlkonsistenz, geringe Krämpfe; Stuhl ≤ 5 mal/Tag; geringer Schleim- oder Blutabgang	**2** deutlich verminderte Stuhlkonsistenz und Bauchkrämpfe; Stuhl > 5mal /Tag; viel Schleim oder zeitweilig Blutabgang	**3** Massiv vermehrte (wäßrige) Stühle, operationsbedürftige Darmobstuktion, (Ileus) oder Blutung	**4** Nekrose; Perforation, Fistel; (sonstige lebensbedrohliche Darmkomplikationen)
**Leber**	**0** normal	**1** Geringe Müdigkeit, Übelkeit, Dyspepsie,	**2** Mäßige Symptomatik (Müdigkeit, Übelkeit, Dyspepsie)	**3** Ausgeprägte Leberinsuffizienz	**4** Lebernekrose, hepatogene(s) Koma oder Enzephalopathie; (parenterale Ernährung, portocavaler Shunt erforderlich)
**Bilirubin**	**0** Altersnorm (N)	**1** > N – 1,5 x N	**2** > 1,5 – 3,0 x N	**3** > 3,0 – 10,0 x N	**4** > 10,0 x N
**S-GOT / S-GPT**	**0** Altersnorm (N)	**1**> N – 2,5 x N	**2** > 2,5 – 5,0 x N	**3** > 5,0 – 20,0 x N	**4** > 20,0 x N
**Niere**<br>Kreatinin (mg%)<br>Kreatinin-Clearence<br>Proteinurie **(g/l)**	**0**<br>Altersnorm (N)<br>> 90<br>keine	**1**<br>> N – 1,5 x N<br>60 – 89<br>< 3	**2**<br>> 1,5 – 3,0 x N<br>40 –59<br>3 – 10,0	**3**<br>> 3,0 – 6,0 x N<br>20 – 39<br>10,0	**4**<br>> 6,0 x N<br>< 19<br>Nephrotisches Syndrom
**Hämaturie**	**0** keine	**1** mikroskopisch	**2** makroskopisch ohne Koageln	**3** makroskopisch mit Koageln	**4** Transfusion erforderlich
**Harnleiter und Blase**	**0** normal	**1** Geringe Schleimhautatrophie; geringe (kleinflächige) Teleangiektasie; (Harndrang gering verstärkt); Mikrohämaturie	**2** Mäßig verstärkter Harndrang; generalisierte Teleangiektasie; zeitweilig Makrohämaturie; (Polyurie: >6 mal /Tag o. < 1 mal / h)	**3** Massiver Harndrang (>1mal / h) und Dysurie; ausgeprägte generalisierte Teleangiektasie (oft mit Petechien); häufig Hämaturie	**4** Nekrose; (Perforation, Fistel) schwere Blasenkontraktur, schwere hämorrhagische Zystitis; (Zystektomie nötig)
**Knochen**	**0** normal	**1** Keine Symptome; keine Wachstumsverzögerung; (gering) reduzierte Knochendichte	**2** Mäßige Schmerzen oder Spannungsgefühl; (mäßige) Wachstumsstörung; irreguläre Knochen-sklerosierung	**3** Ausgeprägte Schmerzen oder Spannungsgefühl; völliger Wachstumsstillstand; dichte Knochensklerotisierung	**4** Nekrose bzw. Osteoradionekrose; Spontanfraktur
**Gelenke**	**0** normal	**1** Geringe Gelenksteife; (keine Symptome); geringe Funktionseinbußen	**2** Mäßige Gelenksteife; zeitweilige oder mäßige Gelenkschmerzen ; mäßige Funktionseinbußen	**3** Ausgeprägte Gelenksteife; ausgeprägte Gelenkschmerzen mit ausgeprägten Funktionseinbußen	**4** Nekrose; vollständige Gelenksteife (Ankylose mit Funktionsverlust)
**Funktion der bestrahlten Extremitäten**	**0** normal	**1** Diskrete Funktionseinbuße, normale tägliche Aktivitäten möglich.	**2** Mäßige Funktionseinbußen bei täglicher Aktivität.	**3** Ausgeprägte Funktionseinbuße.	**4** Funktionsverlust
**Gehirn (ZNS)**	**0** normal	**1** Geringe Kopfschmerzen, geringe Lethargie (geringe neurologische Ausfälle)	**2** Mäßige Kopfschmerzen, deutliche Lethargie (Somnolenz < 50% / Tag); mäßige neurologische Ausfälle	**3** Starke Kopfschmerzen und ausgeprägte ZNS-Störungen, z.B. teilweise Kraftverlust oder Dyskinesie (und Somnolenz > 50%); ausgeprägte neurologische Ausfälle	**4** Krampfanfälle oder Lähmungen, Bewußtlosigkeit (massive oder lebensbedrohliche neurologische Ausfälle)
**Rückenmark (periphere Nerven)**	**0** normal	**1** Diskretes L'hermitte-Zeichen (Parästhesien, reduzierte Reflexe)	**2** Ausgeprägtes L'hermitte-Zeichen (mit Parästhesien oder Muskelschwäche)	**3** Objektive (segmentale) neurologische Ausfälle (mit Parästhesien oder Paresen)	**4** Mono-, Para-, Tetraplegie
**Augen**	**0** normal	**1** Asymptomatische Linsentrübung; geringe Kornea-Ulzeration oder Keratitis (und / oder Konjunktivitis)	**2** Symptomatische Linsentrübung; mäßige Kornea-ulzeration (Keratitis); mäßige Retinopathie oder Glaukom	**3** Ausgeprägte Keratitis (Ulzeration) ausgepröägte Retinopathie mit Netzhautablösung; ausgeprägtes Glaukom	**4** Massive ophthalmologische Störungen; Panophthalmitis; (uni-/ bilateraler Visusverlust
**Ohren**	**0** normal	**1** Geringe Otitis externa mit Atrophie oder Fibrose; keine Therapie; Audiogramm : Hörverlust < 10 dB	**2** Mäßige Otitis externa mit Atrophie oder Fibrose; (Tinnitus); lokale Therapie; mäßige Hypakusis (10 - 15 dB)	**3** Ausgeprägte Otitis externa et media; (ständiger Tinnitus nicht medikamentös bedingt); starke Hypakusis (> 15 - 20 dB)	**4** Schwere Osteochondritis, Ulzeration, Nekrose; vollständige ein-/beidseitige Taubheit (> 20 dB)

Anmerkungen:...

....

Wachstum/Pubertät:

Körperhöhe in cm: ____________ **Sitzhöhe in cm:** ____________

Körpergewicht in kg: ____________

Skoliose: ☐ nein ☐ ja

Deformitäten an Knochen und Weichteilen: ☐ nein ☐ ja, Lokalisation:...

Beschreibung: ....

....

....

Pubertätsentwicklung (nach Schädel-, Becken- oder Oberschenkelbestrahlung; Laborwerte aus der Pädiatrie, bitte Einheit angeben !):

bei Mädchen:Menarche: ☐ nein ☐ ja

Labor: LH: ....
FSH: ....

Tannerstadium Brust:.......................................

Östradiol: ....

Tannerstadium Schambehaarung:...................

Prolaktin: ....

bei Jungen: Tannerstadium männliche Genitale:..................

Labor: LH: ....

Tannerstadium Schambehaarung:.....................

FSH: ....

Hodenvolumen (bei Päd. zu erfragen):..............

Testosteron: ....
Prolaktin: ....

Schilddrüsenwerte (nach Rad. Kopf/Hals/Mediastinum):

TSH basal: __________µE / ml (Werte bei Pädiatern zu erfragen)

fT4: __________ng / dl

fT3: __________ng/dl

Datum:_________________

Aktuelle Schilddrüsen-Medikation:

Präparat:______________________

Dosierung:_____________________

nach ZNS-Bestrahlung:

neurokognitiver Test durchgeführt: ☐ nein ☐ ja
(im Rahmen päd. Nachsorge)

pathologisch: ☐ nein ☐ ja

....
Ausfüllender Arzt

....
Unterschrift/Klinikstempel
